# Responses of carbapenemase-producing and non-producing carbapenem-resistant *Pseudomonas aeruginosa* strains to meropenem revealed by quantitative tandem mass spectrometry proteomics

**DOI:** 10.3389/fmicb.2022.1089140

**Published:** 2023-02-09

**Authors:** Francisco Salvà-Serra, Daniel Jaén-Luchoro, Nachiket P. Marathe, Ingegerd Adlerberth, Edward R. B. Moore, Roger Karlsson

**Affiliations:** ^1^Department of Infectious Diseases, Institute for Biomedicine, Sahlgrenska Academy, University of Gothenburg, Gothenburg, Sweden; ^2^Department of Clinical Microbiology, Sahlgrenska University Hospital, Gothenburg, Sweden; ^3^Culture Collection University of Gothenburg (CCUG), Department of Clinical Microbiology, Sahlgrenska University Hospital and Sahlgrenska Academy, University of Gothenburg, Gothenburg, Sweden; ^4^Centre for Antibiotic Resistance Research (CARe), University of Gothenburg, Gothenburg, Sweden; ^5^Microbiology, Department of Biology, University of the Balearic Islands, Palma de Mallorca, Spain; ^6^Institute of Marine Research (IMR), Bergen, Norway; ^7^Nanoxis Consulting AB, Gothenburg, Sweden

**Keywords:** *Pseudomonas aeruginosa*, carbapenem-resistance, multidrug resistance, nano-LC–MS/MS, tandem mass tag, quantitative shotgun proteomics, extensively-drug resistant, high-risk clones

## Abstract

*Pseudomonas aeruginosa* is an opportunistic pathogen with increasing incidence of multidrug-resistant strains, including resistance to last-resort antibiotics, such as carbapenems. Resistances are often due to complex interplays of natural and acquired resistance mechanisms that are enhanced by its large regulatory network. This study describes the proteomic responses of two carbapenem-resistant *P. aeruginosa* strains of high-risk clones ST235 and ST395 to subminimal inhibitory concentrations (sub-MICs) of meropenem by identifying differentially regulated proteins and pathways. Strain CCUG 51971 carries a VIM-4 metallo-β-lactamase or ‘classical’ carbapenemase; strain CCUG 70744 carries no known acquired carbapenem-resistance genes and exhibits ‘non-classical’ carbapenem-resistance. Strains were cultivated with different sub-MICs of meropenem and analyzed, using quantitative shotgun proteomics based on tandem mass tag (TMT) isobaric labeling, nano-liquid chromatography tandem-mass spectrometry and complete genome sequences. Exposure of strains to sub-MICs of meropenem resulted in hundreds of differentially regulated proteins, including β-lactamases, proteins associated with transport, peptidoglycan metabolism, cell wall organization, and regulatory proteins. Strain CCUG 51971 showed upregulation of intrinsic β-lactamases and VIM-4 carbapenemase, while CCUG 70744 exhibited a combination of upregulated intrinsic β-lactamases, efflux pumps, penicillin-binding proteins and downregulation of porins. All components of the H1 type VI secretion system were upregulated in strain CCUG 51971. Multiple metabolic pathways were affected in both strains. Sub-MICs of meropenem cause marked changes in the proteomes of carbapenem-resistant strains of *P. aeruginosa* exhibiting different resistance mechanisms, involving a wide range of proteins, many uncharacterized, which might play a role in the susceptibility of *P. aeruginosa* to meropenem.

## 1. Introduction

*Pseudomonas aeruginosa* is an adaptable and widely-distributed Gram-negative bacterium, which is found largely in environments associated with human activity (Crone et al., 2019; Diggle and Whiteley, 2020) and that can cause a wide range of opportunistic infections (Pelegrin et al., 2021). *P. aeruginosa* is one of the leading causes of severe nosocomial infections, including ventilator-associated pneumonia, the most common infection among intensive care unit patients (Bassetti et al., 2012). It is a major cause of chronic respiratory infections in patients with cystic fibrosis or other underlying conditions such as bronchiectasis or chronic obstructive pulmonary disease (Döring et al., 2011; Garcia-Clemente et al., 2020; Vidaillac and Chotirmall, 2021).

*P. aeruginosa* is one of the most problematic drug-resistant pathogens and treatment options are often challenged by the increasing emergence of multidrug and extensively drug-resistant strains (Potron et al., 2015; Bassetti et al., 2018), which are associated with increased morbidity, as well as mortality (Hirsch and Tam, 2010; Morata et al., 2012; Micek et al., 2015). Indeed, *P. aeruginosa* is among the six leading pathogens causing deaths associated with antimicrobial resistance (Murray et al., 2022). Carbapenem antibiotics, such as meropenem, still remain active and effective against many multidrug-resistant isolates (Bassetti et al., 2018). Carbapenems are β-lactam antibiotics and thus they inhibit cell wall biosynthesis by entering the periplasmatic space and binding to penicillin-binding proteins (PBPs), which are involved in the synthesis of peptidoglycan (Kitzis et al., 1989; Yang et al., 1995). Compared with other β-lactams such as penicillins and cephalosporins, carbapenems have broader activity spectrum, are less susceptible to hydrolysis by β-lactamases and, in some cases, act as β-lactamase inhibitors; therefore, they are frequently used as “last-resort antibiotics” (Papp-Wallace et al., 2011; Meletis, 2016). However, the frequency of infections caused by *P. aeruginosa* strains resistant to carbapenems is increasing worldwide (Potron et al., 2015); the World Health Organization (WHO) included carbapenem-resistant *P. aeruginosa* in the Priority 1 (critical) group of the list of priority pathogens for which research and development of new antibiotics is urgently needed (Tacconelli et al., 2018).

In *P. aeruginosa*, carbapenem-resistance is often driven by an interplay of well-known intrinsic, acquired and adaptive resistance mechanisms, that are typically classified into three broad categories: drug transport, drug inactivation and target modification (Blair et al., 2015). These include, for instance, a naturally low outer membrane permeability, mutations leading to truncated outer membrane porins or over-expression of efflux pumps, presence of naturally-occurring and horizontally-acquired β-lactamases, and regulation or alteration of PBPs (Rodríguez-Martínez et al., 2009b; Pang et al., 2019).

However, the mechanisms behind carbapenem resistance are not always obvious (Johnning et al., 2018; Cortes-Lara et al., 2021) and, for instance, mutations in core metabolic genes of *E. coli* recently have been shown to confer resistance to various antibiotics, and to be more common than previously thought. This strengthens the hypothesis that alternative non-canonical resistance mechanisms can play important roles in drug resistance (Lopatkin et al., 2021). In fact, the presence/absence of dozens of genes, including genes involved in metabolism, have been shown to alter the susceptibility to β-lactam antibiotics in *P. aeruginosa* (Alvarez-Ortega et al., 2010). Thus, multiple individual low-level resistance mechanisms that would not be significant on their own, collectively may contribute to the development of clinically-relevant levels of resistance. Unveiling the unknown resistance factors is essential for gaining a deeper understanding of carbapenem resistance in bacteria (Baquero, 2001). Furthermore, this complex interplay is enhanced by the remarkably large regulatory network of *P. aeruginosa* and its high degree of responsive capacity to environmental stimuli (Stover et al., 2000; Moradali et al., 2017).

In this context, applying non-targeted open approaches, such as shotgun methodologies, is essential to understand the complexity of resistance mechanisms and the strains, global responses, as well as to identify possible novel mechanisms and pathways involved in the response and resistance to antibiotics. Among those, quantitative proteomic techniques have the capacity to determine variations between conditions in the relative abundance of thousands of proteins and, therefore, such approaches have potential to reveal mechanisms involved in the responses to antibiotics that would not be possible to elucidate using classical methods of resistance detection (Park et al., 2016; Pérez-Llarena and Bou, 2016).

The aim of this study was to identify responses of carbapenem-and extensively drug-resistant clinical *P. aeruginosa* strains exposed to varying sub-lethal concentrations of meropenem. We further identified proteins with changes in their relative abundances (i.e., proteins with upregulated or downregulated abundance levels; from now on, referred to as, “upregulated,” “downregulated” or “differentially regulated” proteins), groups of proteins and pathways potentially implicated in carbapenem resistance and the responses to sub-minimal inhibitory concentrations (sub-MICs) of meropenem of carbapenemase-producing (i.e., “classical” carbapenem resistance) and carbapenemase non-producing (i.e., “non-classical” carbapenem resistance) *P. aeruginosa* strains. For that, a “bottom-up” quantitative shotgun proteomic approach, using tandem mass tags (TMT) peptide labeling (Thompson et al., 2003), followed by nano-liquid chromatography tandem-mass spectrometry (nano-LC–MS/MS) was applied. Although previous studies have analyzed the effects of β-lactams (including carbapenems) and other antibiotics on global gene expression of *P. aeruginosa* (Bagge et al., 2004; Blázquez et al., 2006; Morita et al., 2014), this is the first study applying a quantitative shotgun proteomics approach to determine the global proteomic responses of carbapenemase-producing and non-producing carbapenem-resistant strains of *P. aeruginosa*, belonging to high-risk clones, on exposure to sub-lethal concentrations of meropenem.

## 2. Materials and methods

### 2.1. Overview of the methodology

**Figure 1:**
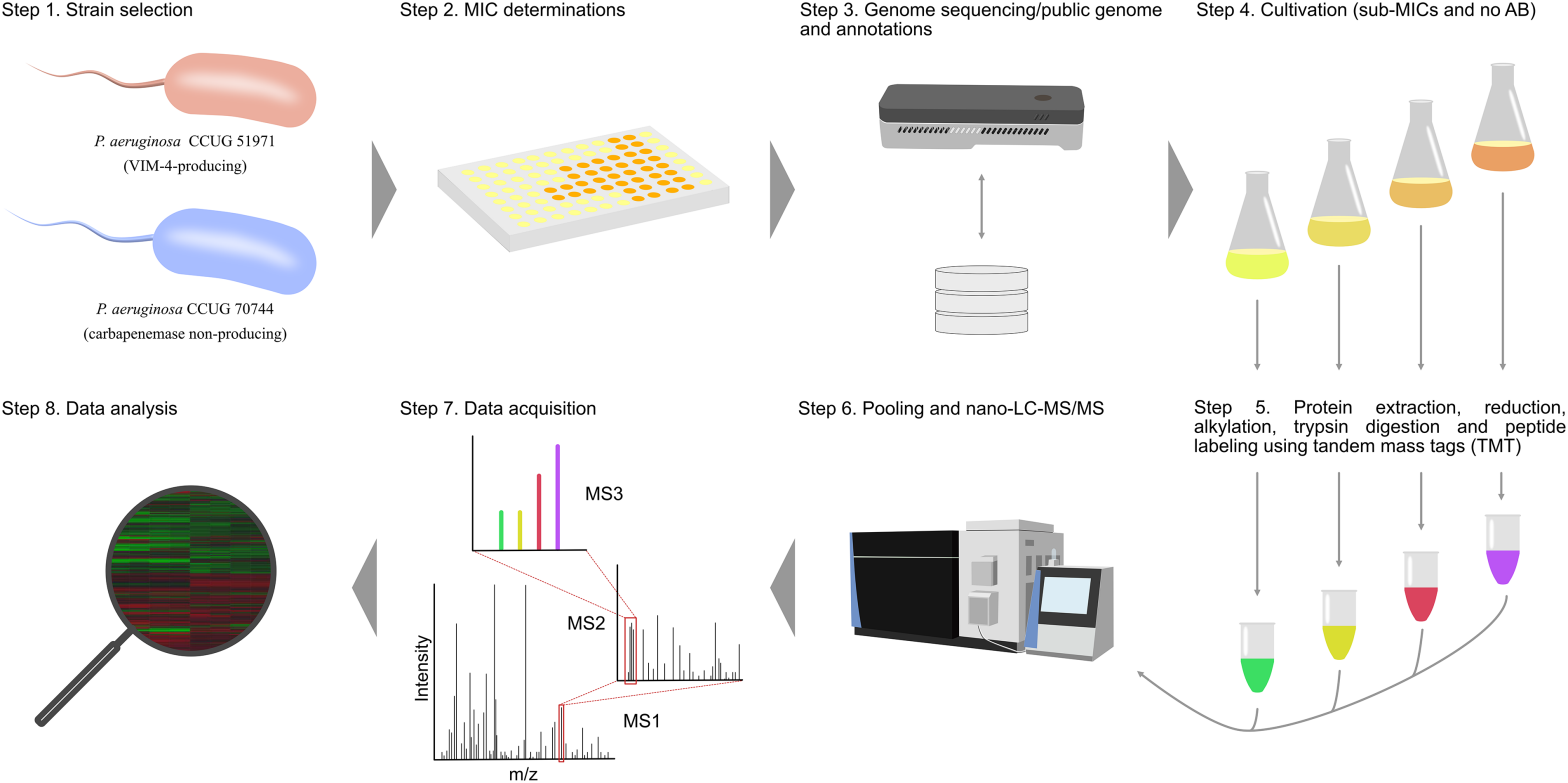
Experimental setup of the study. Each strain was cultivated with three different sub-MICs of meropenem and without antibiotic. Subsequently, proteins were extracted, reduced, alkylated and digested with trypsin. Peptides were labeled, using two TMT sets, pooled and analyzed, using nano-LC–MS/MS for protein detection and relative quantitation.

Two carbapenem-resistant *P. aeruginosa* strains, one with a “classical” carbapenemase-dependent resistance (CCUG 51971) and one with a “non-classical,” undefined resistance (CCUG 70744) were cultivated with three different sub-MIC levels of meropenem and without antibiotic. Subsequently, proteins were extracted, reduced, alkylated and digested with trypsin, with the resulting peptides labeled, using TMTs (Thompson et al., 2003). For each strain, two TMT 11-plex sets were used and four technical replicates per condition were made (two in each TMT set). *P. aeruginosa* CCUG 56489 (= PAO1), was cultivated without antibiotic and included as a control in each TMT set. The labeled samples of each set were combined and analyzed by nano-LC–MS/MS. Proteins were identified and their relative abundances determined by comparing the protein expression levels at sub-MICs and no antibiotic conditions (Figure 1).

### 2.2. Bacterial strains

Strain CCUG 51971 (=PA 66; sequence type 235), isolated from a human urine sample, at the Karolinska Hospital (Stockholm, Sweden; Giske et al., 2003), carries a class-1 integron-encoded VIM-4 metallo-β-lactamase (MBL), responsible for the high carbapenem resistance levels (MIC of imipenem and meropenem >256 μg/ml; MIC of imipenem + ethylene-diaminetetraacetic acid [EDTA] = 6 μg/ml; Giske et al., 2003; Yoon and Jeong, 2021). CCUG 51971 was the first described MBL-producing *P. aeruginosa* isolate in Scandinavia (Giske et al., 2003). Strain CCUG 70744 (sequence type 395), isolated from a human sputum sample, at the Sahlgrenska University Hospital (Gothenburg, Sweden), carries no known acquired antibiotic resistance genes (Johnning et al., 2018). Additionally, strain CCUG 56489 (= PAO1) was included as a carbapenem-susceptible control. Details of the strains are presented in Table 1.

**Table 1:** General characteristics of the strains and minimal inhibitory concentrations (MICs) of the antimicrobial agents included in the “*Pseudomonas*/*Acinetobacter* standard panel” of the National Reference Laboratory for Antibiotic Resistance (Växjö, Sweden).

Section	Features/antibiotic	*Pseudomonas aeruginosa* CCUG 56489 (= PAO1)	*Pseudomonas aeruginosa* CCUG 51971 (= PA 66)	*Pseudomonas aeruginosa* CCUG 70744
General information	Isolation source	Human infected wound	Human urine	Human sputum
	Geographical origin	Australia	Stockholm, Sweden	Gothenburg, Sweden
	Collection date	Before 1955	2001–11	2013-01-08
	NCBI RefSeq accession number	NC_002516.2	NZ_CP043328.1	NZ_CP023255.1
	Genome sequence length (bp)	6,264,404	7,012,798	6,859,232
	Number of replicons	1	1	1
	Protein sequences^a^	5,572	6,490	6,399
	ANIb (%)^b^	99.16	98.68	99.20
	Sequence type	549	235	395
MIC (μg/ml)	Amikacin (aminoglycoside)	2 (S)	**>32 (R)**	16 (S)
	Aztreonam (monobactam)	4 (I)	**64 (R)**	**>64 (R)**
	Cefepime (cephalosporin)	2 (I)	**>32 (R)**	**>32 (R)**
	Ceftazidime (cephalosporin)	2 (I)	**>32 (R)**	**>32 (R)**
	Ciprofloxacin (fluoroquinolone)	≤0.12 (I)	**>8 (R)**	**8 (R)**
	Colistin (peptide antibiotic)	1 (S)	1 (S)	**8 (R)**
	Gentamicin (aminoglycoside)	1 (IE)	>16 (IE)	8 (IE)
	Imipenem (carbapenem)	4 (I)	**>32 (R)**	**16 (R)**
	Levofloxacin (fluoroquinolone)	0.5 (I)	**>8 (R)**	**8 (R)**
	Meropenem (carbapenem)	1 (S)	**512 (R)**	**16 (R)**
	Piperacillin-tazobactam (penam)	8 (I)	**>64 (R)**	**>64 (R)**
	Tobramycin (aminoglycoside)	0.5 (S)	**>16 (R)**	2 (S)
	Trimethoprim-sulfamethoxazole (diaminopyrimidine antibiotic)	**8 (R)**	**>16 (R)**	**4 (R)**

The drug class of each antibiotic is indicated in parenthesis. EUCAST breakpoint categories are indicated in parenthesis. S, Susceptible, standard dosing regimen (green highlighting); I, Susceptible, increased exposure (yellow highlighting); R, Resistant (bold and red highlighting); IE, Insufficient evidence that the organism or group is a good target for therapy with the agent (gray highlighting).

a Accessioned protein products annotated on the genome sequences.

b ANIb with *Pseudomonas aeruginosa* DSM 50071^T^ (GenBank accession number: CP012001.1).

Lyophiles of the three strains were obtained from the Culture Collection University of Gothenburg (CCUG, Gothenburg, Sweden; www.ccug.se). The strains were reconstituted on Mueller-Hinton agar (Substrate Unit, Department of Clinical Microbiology, Sahlgrenska University Hospital), at 37°C, for 24 h.

### 2.3. Antibiotic susceptibility testing

Minimal inhibitory concentrations (MIC) were determined at the National Reference Laboratory for Antibiotic Resistance (Växjö, Sweden; http://www.mikrobiologi.org/referenslaboratorium), using the broth dilution method, in accordance with the EUCAST (European Committee on Antimicrobial Susceptibility Testing) recommendations and the ISO standard 20776-1 (2006). The MICs of 13 antimicrobial agents included in the “*Pseudomonas*/*Acinetobacter* standard panel” (analysis no. 25630) were determined. Additionally, strain CCUG 51971 was tested in-house for higher concentrations of meropenem, following the same recommendations. Clinical breakpoints were set, according to the EUCAST breakpoint tables v12.0 (2022).^1^

### 2.4. DNA extraction and whole-genome sequencing

High-molecular weight genomic DNA of *P. aeruginosa* CCUG 51971 was obtained from fresh biomass, using a previously described protocol (Salvà-Serra et al., 2018). DNA integrity was verified on a TapeStation 2200 instrument (Agilent Technologies Inc., Santa Clara, CA, United States), using a Genomic DNA ScreenTape and Reagents kit (Agilent Technologies Inc.). Subsequently, isolated genomic DNA was used to prepare a standard Illumina library at Eurofins Genomics (Konstanz, Germany), with insert sizes ranging from 130 to 680 bp, following an optimized protocol and using standard Illumina adapter sequences. The genomic DNA library was sequenced, using an Illumina NovaSeq 6000 system (Illumina, Inc., San Diego, CA, United States), to generate paired-end reads of 151 bp. Genomic DNA also was used to prepare an Oxford Nanopore sequencing library, using a Rapid Barcoding Kit (SQK-RBK004; Oxford Nanopore Technologies, Ltd., Oxford, United Kingdom), following the manufacturer’s protocol. The library was sequenced on a MinION Mk1B device (Oxford Nanopore Technologies, Ltd.), using a Flow Cell FLO-MIN106 vR9.4 and the software MinKNOWN v19.05 (Oxford Nanopore Technologies, Ltd.), with the 48-h sequencing script and default parameters.

### 2.5. Genome assembly and annotation

The quality of the Illumina reads was analyzed, using CLC Genomics Grid Worker v11.0.3 (Qiagen Aarhus A/S, Aarhus, Denmark). The Oxford Nanopore reads, in raw FAST5 format, were base-called, using Guppy v3.1.5 (Oxford Nanopore Technologies, Ltd.), and the quality of the sequence reads analyzed, using NanoPlot v1.26.3 (De Coster et al., 2018). Subsequently, all Illumina and Nanopore reads were assembled *de novo*, using Unicycler v0.4.7 (Wick et al., 2017). The assembly was evaluated, using QUAST v5.0.2 (Gurevich et al., 2013), submitted to GenBank (Sayers et al., 2020) and annotated, using the NCBI Prokaryotic Genome Annotation Pipeline (PGAP) v4.9 (Tatusova et al., 2016). The publicly available complete genome sequences of *P. aeruginosa* PAO1 (Stover et al., 2000) and *P. aeruginosa* CCUG 70744 (Johnning et al., 2018) were downloaded from the NCBI Reference Sequence (RefSeq) database (O'Leary et al., 2016). To confirm the species identity of the strains, average nucleotide identity values based on BLAST (ANIb; Goris et al., 2007) were calculated, using JSpeciesWS (Richter et al., 2016), between the genome sequences of the three strains and that of the type strain of *P. aeruginosa* (DSM 50071^T^; GenBank accession number: CP012001.1). Multilocus sequence typing (MLST) of the complete genome sequences was performed, using the tool MLST v2.0.4 and the MLST database v2.0.0 of the Center for Genomic Epidemiology (CGE; Larsen et al., 2012), with the *P. aeruginosa* MLST profile (Curran et al., 2004).

### 2.6. Cultivation conditions for the quantitative proteomics experiments

Preinocula were prepared in 4 ml of Mueller-Hinton broth, incubated, at 37°C, overnight, with orbital shaking (250 rpm). Cells were pelleted by centrifugating at 10,000 × *g*, for 5 min, and resuspended in phosphate-buffered saline (PBS) adjusting the turbidity to MacFarland Standard 0.5 (equal to 1–2 × 10^8^ colony forming units, CFUs/ml; Wiegand et al., 2008). Subsequently, 250 μl were inoculated in baffled Erlenmeyer flasks of 250 ml capacity containing Mueller-Hinton broth, to a final volume of 50 ml. The flasks were incubated, at 37°C, for 20 h, with orbital shaking (250 rpm). Strain CCUG 56489 (= PAO1) was cultivated without antibiotic. Strain CCUG 70744 was cultivated with 0, 2, 4 (¼ of MIC) and 8 μg/ml (½ of MIC) of meropenem (Sigma-Aldrich, St. Louis, MO, United States). Strain CCUG 51971 was cultivated with 0, 8, 128 (¼ of MIC) and 256 μg/ml (½ of MIC) of meropenem.

### 2.7. Peptide generation and TMT labeling

Bacterial biomasses were harvested by centrifugation (10,000 × *g*, 5 min), and washed three times with 1.0 ml of PBS, by discarding the supernatant, resuspending the pellet and centrifuging at 10,000 × *g*, for 5 min. Washed bacteria were resuspended in PBS, to optical density 1.0 at a wavelength of 600 nm (OD_600_), and divided into four tubes (four technical replicates for nano-LC–MS/MS). Bacterial cells were pelleted by centrifugation at 10,000 × *g*, for 5 min, resuspended in 150 μl of PBS plus 15 μl of 20% sodium dodecyl sulfate (SDS) and transferred to small vials (200 μl) containing acid-washed glass beads (diameter: 150–212 μm; Sigma-Aldrich). The cells were lysed by bead-beating, using a TissueLyser II (Qiagen, Hilden, Germany), at 25 Hz, for 5 min, and the lysates were frozen at −20°C until analysis.

Protein concentrations were determined using a Pierce™ BCA Protein Assay kit (Thermo Fisher Scientific, Waltham, MA, United States) and a Benchmark Plus Microplate Reader (Bio-Rad, Hercules, CA, United States). Bovine serum albumin solutions were used as standards. Representative references containing equal amounts from each group were prepared. Proteins were digested with trypsin, using the filter-aided sample preparation (FASP) method (Wiśniewski et al., 2009). Aliquots containing 30 μg of protein from each sample and the references were used for digestions. Briefly, samples were reduced using 100 mM dithiothreitol, at 60°C, for 30 min, transferred to 30 kDa MWCO Pall Nanosep^®^ centrifugation filters (Sigma-Aldrich), washed repeatedly using urea (8 M) and once using digestion buffer (1% sodium deoxycholate [SDC], in 50 mM triethylammonium bicarbonate, TEAB), and alkylated using 10 mM methyl methanethiosulfonate, in digestion buffer, for 30 min. Trypsin digestion was performed in digestion buffer, by adding 0.5 μg of Pierce MS grade Trypsin (Thermo Fisher Scientific) and incubating at 37°C, overnight. An additional portion of trypsin was added and incubated for two additional hours. Peptides were collected by centrifugating, at 10,000 × *g*.

Peptides were labeled, using TMT 11-plex isobaric mass tagging reagents (Thermo Fisher Scientific), following the manufacturer’s instructions. Samples were combined into two TMT-sets with two samples from each group and a reference. SDC was removed by acidifying using 10% trifluoroacetic acid. The combined sets were pre-fractionated into 20 fractions by performing basic reversed-phase liquid chromatography (bRP-LC), using a Dionex UltiMate 3,000 UHPLC system (Thermo Fisher Scientific) and a reversed-phase XBridge BEH C18 column (3.5 μm, 3.0 × 150 mm, Waters Corporation, Milford, MA, United States). For that, a linear gradient was created by mixing solvent A (10 mM ammonium formate buffer, pH 10.0) and solvent B (90% acetonitrile and 10% 10 mM ammonium formate, pH 10.0) at a flow rate of 400 μl/min, increasing solvent B from 3% to 40%, over 18 min, followed by an increase to 100% of solvent B, over 5 min. The fractions were concatenated into 10 fractions (1 + 11, 2 + 12, … 10 + 20), dried and reconstituted in 3% acetonitrile and 0.2% formic acid.

### 2.8. Nano-LC–MS/MS

The fractions were analyzed using an Orbitrap Fusion™ Lumos™ Tribrid™ mass spectrometer coupled with an Easy-nLC1200 nano-liquid chromatography system (Thermo Fisher Scientific). Fractioned peptides were trapped on an Acclaim Pepmap 100 C18 trap column (100 μm × 2 cm, particle size 5 μm, Thermo Fischer Scientific) and separated in an in-house-packed analytical column (75 μm × 30 cm, particle size 3 μm, Reprosil-Pur C18, Dr. Maisch HPLC, Ammerbuch, Germany), using a linear gradient of solvent B (80% acetonitrile, 0.2% formic acid) over solvent A (0.2% formic acid) from 5% to 33%, over 77 min, followed by an increase to 100% of solvent B for 3 min, and 100% solvent B, for 10 min, at a flow rate of 300 nl/min. Precursor ion mass spectra were acquired at 120,000 resolution and MS/MS analysis was performed in a data-dependent multi-notch mode, wherein collision-induced dissociation (CID) spectra of the most intense precursor ions were recorded in the ion trap at a collision energy setting of 35 for 3 s (“top speed” setting). Precursors were isolated in the quadrupole using a 0.7 m/z isolation window. For fragmentation, charge states 2 to 7 were selected. Dynamic exclusion was set to 45 s and 10 ppm. For reporter ion quantitation, MS^3^ spectra were recorded at 50,000 resolution with higher-energy collisional dissociation (HCD) fragmentation at collision energy of 65, using the synchronous precursor selection.

### 2.9. Proteomic data analysis

The data files for each TMT set were merged for identification and relative quantitation, using Proteome Discoverer v2.4 (Thermo Fisher Scientific). The searches were done against the accessioned protein products annotated on the genome sequences (i.e., FAA format files) of *P. aeruginosa* strain CCUG 56489 (= PAO1; RefSeq accession number: NC_002516.2) and *P. aeruginosa* strain CCUG 51971 (RefSeq accession number: NZ_CP043328.1) and *P. aeruginosa* CCUG 70744 (RefSeq accession number: NZ_CP023255.1), using Mascot v2.5.1 (Matrix Science, London, United Kingdom) as a search engine and both databases defined in the Protein Marker node. The precursor mass tolerance was set to 5 ppm and fragment mass tolerance to 0.6 Da. Tryptic peptides were accepted with zero missed cleavage, variable modifications of methionine oxidation and fixed cysteine alkylation, TMT-label modifications of N-terminal and lysine were selected. The reference samples were used as denominator and for calculation of the ratios. Percolator was used for the validation of identified proteins. TMT reporter ions were identified in the MS^3^ HCD spectra with 3 mmu mass tolerance, and the TMT reporter intensity values for each sample were normalized on the total peptide amount. The quantified proteins were filtered at 1% false discovery rate and grouped by sharing the same sequences to minimize redundancy. Unique and razor peptides for a given protein were considered for quantification of the proteins.

### 2.10. Statistical analysis

Heatmaps and principal component analysis (PCA) plots were created in R v4.0.3. The expression values were log2-transformed. The heatmaps were generated, using the *pheatmap* function. The PCAs were done using the *prcomp* function of the stats package, and plotted, using *ggplot2*.

Protein relative abundances were calculated by comparing the samples from the cultures exposed to sub-MICs of meropenem with those from the cultures exposed to no antibiotic. Only proteins with, at least, two peptide matches were considered. A Welch’s *t*-test (i.e., unequal variances *t*-test) was performed in Microsoft Excel (four technical replicates for each sub-MIC vs. four technical replicates without antibiotic). Proteins with fold changes equal to or higher than ±1.5 and *p*-value < 0.05 were considered proteins with significantly different abundances (i.e., proteins with significantly upregulated or downregulated abundance levels, referred to as, “upregulated” or “downregulated” proteins). Clusters of significantly upregulated or downregulated proteins overlapping between conditions were calculated and visualized, using BioVenn (Hulsen et al., 2008). For each pairwise comparison, the fold change and the *p*-value were used to generate a volcano plot [log2(fold change) vs. −log10(*p*-value)], using Microsoft Excel. Additionally, the samples from the cultures exposed to no antibiotic were compared with the samples from strain CCUG 56489 (= PAO1) to determine differences in basal protein abundances. For that inter-strain comparison, only homologs proteins sharing ≥99% of sequence identity and with 100% coverage over the longer protein were considered. The clustering and homology filtering was performed, using Cd-hit-2d (Li and Godzik, 2006) at CD-HIT Suite (Huang et al., 2010).

### 2.11. Functional annotations and enrichment analyses

The accessioned protein products annotated on the genome sequences and available in RefSeq (O'Leary et al., 2016) were compared with the those of strain PAO1 using Cd-hit-2d (Li and Godzik, 2006) at CD-HIT Suite (Huang et al., 2010). Information from the *Pseudomonas* Genome Database (Winsor et al., 2016) was added to those proteins sharing ≥95% of sequence identity and ≥95% coverage of the longest protein. The proteins were further annotated and assigned to Gene Onthology (GO) terms (Ashburner et al., 2000; Gene Ontology Consortium, 2020), using Blast2GO (Conesa et al., 2005) implemented in OmicsBox v1.3.3 (BioBam Bioinformatics S.L., Valencia, Spain). They were also annotated using InterProScan v5.54–87.0 (Jones et al., 2014) and eggNOG-Mapper v2.1.0 (Huerta-Cepas et al., 2017) with eggNOG v5.0.2 (Huerta-Cepas et al., 2019) implemented in OmicsBox v2.0.36. eggNOG mapper annotation transfers were limited to terms with experimental evidence and one-to-one orthology (i.e., prioritizing precision). Subsequently, the GO annotations were validated to remove all redundant terms based on the GO True Path Rule and taxonomically filtered, using the class *Gammaproteobacteria* (Taxonomy ID: 1236). The remaining GO terms were mapped to Enzyme Commission (EC) numbers. KEGG (Kyoto Encyclopedia of Genes and Genomes) pathways were annotated, using OmicsBox v2.0.36, *via* eggNOG and the assigned EC numbers (Kanehisa et al., 2020). The accession protein products also were annotated and classified into Clusters of Orthologous Groups (COG) categories (Galperin et al., 2015), using eggNOG-Mapper v2 (Huerta-Cepas et al., 2017), with the eggNOG v5.0 orthology resource (Huerta-Cepas et al., 2019) and the conditions listed above.

Antibiotic resistance genes were searched by analyzing the accessioned protein products, using the tool, Resistance Gene Identifier (RGI) v5.1.1 of the Comprehensive Antibiotic Resistance Database (CARD) v3.0.7 (Alcock et al., 2020), limiting the results to “perfect” and “strict” hits only. β-lactamase types and variants were confirmed, using the Beta-Lactamase DataBase (Naas et al., 2017). Regulatory proteins were predicted, using the webserver P2RP v2.7 (Barakat et al., 2013). Putative integrative and conjugative elements (ICE) were searched, using the on-line tool ICEfinder v1.0 (Liu et al., 2018). Type VI secretion systems (T6SSs) were predicted, using SecReT6 v3.0 (Li et al., 2015). Pfam families were searched using the HMMER webserver v2.40 (Potter et al., 2018; Mistry et al., 2021). Additional functional analyses also were done, using the InterPro webserver (Blum et al., 2020). For accuracy, the annotation and information of certain proteins were checked in the *Pseudomonas* Genome Database (Winsor et al., 2016), wherein orthologue searches were performed, using DIAMOND, with default parameters (i.e., query coverage and identity cutoffs: 70%, E-value: 1 × 10^−12^; Buchfink et al., 2015). Frameshifted genes were confirmed by Illumina read mapping and manual examination, using CLC Genomics Grid Worker v11.0.3 (Qiagen Aarhus A/S) with default parameters.

To test if a COG category contained a significantly higher proportion of upregulated or downregulated proteins and to test if any category was significantly enriched, using all quantitated proteins as a background, a Fisher’s exact test was performed, and the Benjamini-Hochberg procedure was used to control for multiple testing. Gene Set Enrichment Analyses (GSEA) of the assigned GO terms and KEGG pathways were performed, using OmicsBox v2.0.36. The enrichment statistic was “weighted (*p* = 1),” and 1,000 permutations were performed. The maximum and minimum gene set sizes were 500 and 15, respectively (default). Only sets with a false discovery rate (FDR) *q*-value <0.05 were deemed significantly enriched. The results were displayed in RStudio v2021.09.2, using *ggplot2* in R v4.1.2, following a previously described protocol (Bonnot et al., 2019).

## 3. Results and discussion

Carbapenem-resistant *P. aeruginosa* strains are a major threat to human health (Tacconelli et al., 2018; Murray et al., 2022) and numerous prominent intrinsic, acquired, and adaptive mechanisms of carbapenem-resistance have been described (Rodríguez-Martínez et al., 2009b; Pang et al., 2019). However, the mechanisms behind carbapenem resistance are not always clear (Johnning et al., 2018; Cortes-Lara et al., 2021), and multiple non-canonical, low-level resistance mechanisms might contribute toward the development of clinically significant levels of resistance (Baquero, 2001; Alvarez-Ortega et al., 2010; Lopatkin et al., 2021).

In this study, we applied a quantitative shotgun proteomic approach to study the global effect of sub-MICs of meropenem on one carbapenemase-producing and one non-carbapenemase-producing carbapenem and extensively drug-resistant *P. aeruginosa* strains, both belonging to high-risk clones: sequence type (ST) 235, the most prevalent worldwide (Oliver et al., 2015), and ST395, respectively.

### 3.1. Antibiotic susceptibility testing

Strain CCUG 51971 encodes a VIM-4 MBL, which confers high levels of resistance to imipenem and meropenem (Giske et al., 2003). *Pseudomonas aeruginosa* CCUG 70744 does not harbor any known acquired carbapenemase gene but exhibited clinically significant levels of phenotypic carbapenem resistance (Johnning et al., 2018). Antibiotic susceptibility testing confirmed the high level of carbapenem resistance of strain CCUG 51971 (MIC of imipenem: >32 μg/ml; MIC of meropenem: 512 μg/ml) and the clinically significant resistance levels of strain CCUG 70744 (MIC of imipenem: 16 μg/ml; MIC of meropenem: 16 μg/ml). Additionally, resistance to multiple other antibiotics belonging to various classes confirmed that both strains can be classified as extensively drug-resistant, as previously defined (Magiorakos et al., 2012; Table 1).

### 3.2. Whole-genome sequencing and multilocus sequence typing

Illumina sequencing of *P. aeruginosa* CCUG 51971 produced 12,783,454 paired-end reads of 151 bp (i.e., 1.93 Gb) with an average Phred quality score of 35.8. Oxford Nanopore sequencing generated 584,423 reads with an average sequence read length of 9,258 bp (i.e., 5.41 Gb), an N50 of 18,815 bp and an average Phred quality score of 9.5.

*De novo* assembly of all sequence reads produced a complete and closed circular chromosome of 7,012,798 bp with a G + C content (mol%) of 66.06%. No plasmids were detected. Annotation revealed 6,773 genes, including 83 RNA genes, 96 pseudogenes and 6,594 protein coding sequences. The ANIb between the genome sequences of strains CCUG 51971, CCUG 70744, CCUG 56489 (= PAO1) and that of *P. aeruginosa* DSM 50071^T^ ranged from 98.68% to 99.20%, confirming their species identity (Table 1). MLST analysis revealed that *P. aeruginosa* CCUG 51971 and CCUG 70744 belong to two high-risk clones: ST235 and ST395, respectively.

### 3.3. Subminimal inhibitory concentrations of meropenem induce extensive changes in the proteome

To evaluate the effect of different sub-MICs of meropenem at the proteome level, the carbapenem-resistant strains *P. aeruginosa* CCUG 51971 and CCUG 70744 were cultivated with three different concentrations of meropenem (CCUG 51971 with 8, 128, and 256 μg/ml; CCUG 70744 with 2, 4, and 8 μg/ml) and without antibiotic. After sample processing and protein digestion, peptides were labeled with TMTs and analyzed using nano-LC–MS/MS.

The analysis detected 3,603 proteins (55.5% of the accessioned protein sequences) in *P. aeruginosa* CCUG 51971; of those, 90.8% (3,271 proteins) were quantitated. In *P. aeruginosa* CCUG 70744, 3,512 proteins were detected (54.9% of the accessioned protein sequences); of those, 92.9% (3,263 proteins) were quantitated (Supplementary Table 1). All detected proteins (i.e., proteins with >1 peptide matches) are listed in Supplementary Tables 2, 3, together with their relative abundances (if quantitated) and functional annotations.

Heatmaps and PCA plots (Supplementary Figures 1, 2) revealed that cultures of both strains exposed to the lowest sub-MIC and to no antibiotic, demonstrated similar protein expression levels. In both cases, the samples from the cultures exposed to the highest sub-MIC demonstrated the highest difference in protein expression levels.

**Figure 2:**
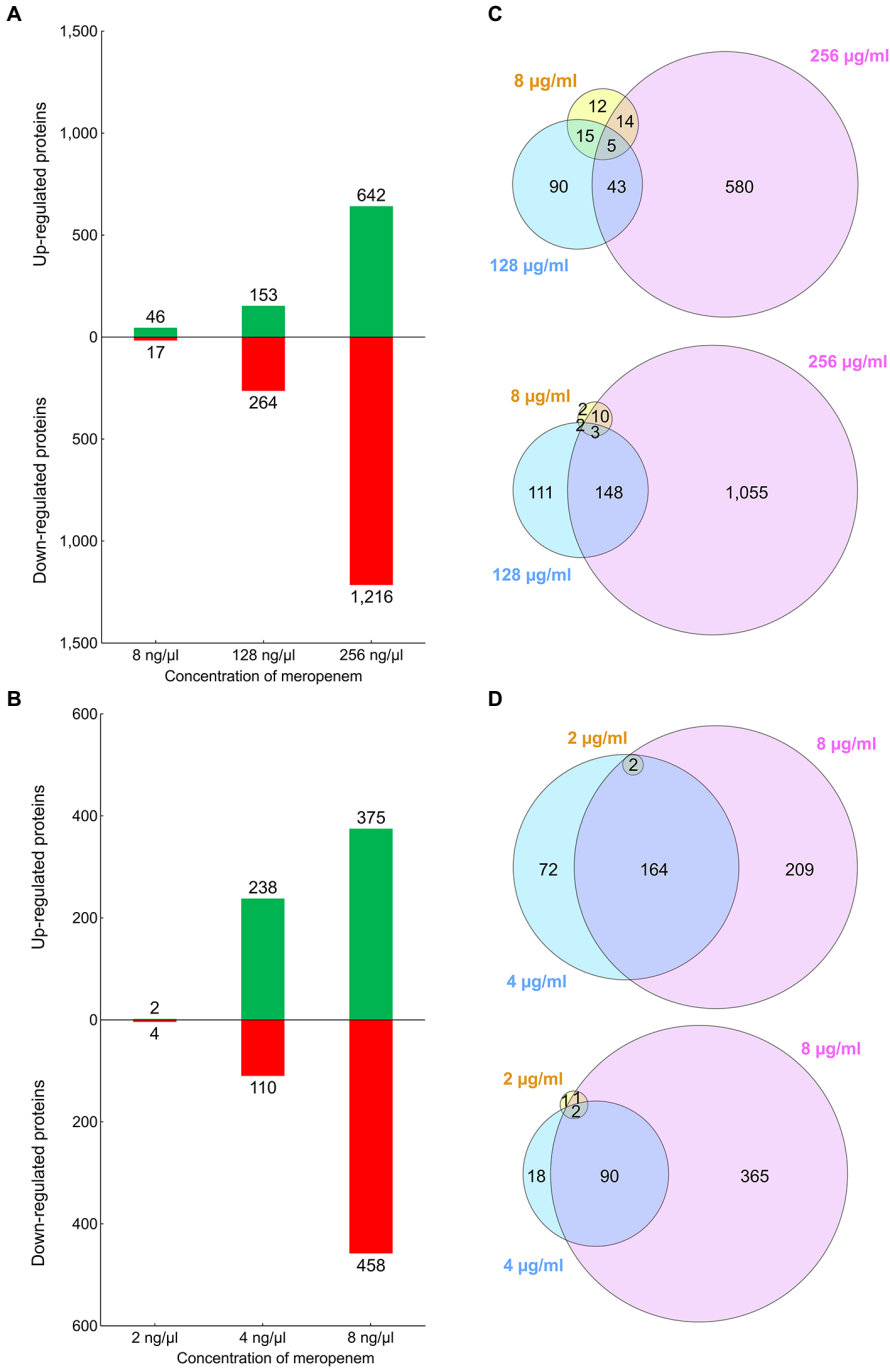
**(A)** Bar graph showing the number of proteins of *Pseudomonas aeruginosa* CCUG 51971 upregulated or downregulated in each condition. **(B)** Bar graph showing the number of proteins of *P. aeruginosa* CCUG 70744 upregulated or downregulated in each condition. **(C)** Venn diagram showing the number of upregulated or downregulated proteins overlapping between conditions in strain CCUG 51971. **(D)** Venn diagram showing the number of upregulated or downregulated proteins overlapping between conditions in strain CCUG 70744.

The relative abundances of the identified proteins were analyzed, by comparing the samples from the cultures exposed to sub-MICs of meropenem, with those from the cultures exposed to no antibiotic. Samples from cultures exposed to higher concentrations of meropenem, had higher numbers of differentially regulated proteins and, on average, higher fold changes (Figure 2; Supplementary Figure 3). In both cases, hundreds of proteins were upregulated and, concomitantly, hundreds downregulated when the strains were cultivated with the highest sub-MICs (i.e., CCUG 51971 with 256 μg/ml and CCUG 70744 with 8 μg/ml, which in both cases is ½ of MIC). Hundreds of proteins were also differentially regulated in each strain when cultivated with 128 and 4 μg/ml, respectively (i.e., ¼ of MIC), while fewer were observed for the lowest concentrations, especially in the case of *P. aeruginosa* CCUG 70744, where only six proteins were differentially regulated at 2 μg/ml. However, this does not come as a surprise, since *P. aeruginosa* has an exceptionally large regulatory network. For instance, *P. aeruginosa* PAO1 harbors 521 genes (9.4% of all annotated genes) encoding transcriptional regulators or two-component systems, which reflects its high versatility and adaptability (Stover et al., 2000). Additionally, β-lactam antibiotics have been previously shown to affect global gene expression in *P. aeruginosa* (Bagge et al., 2004; Blázquez et al., 2006). Other studies performing quantitative shotgun proteomic analyses of *P. aeruginosa*, in those cases exposed to hypoxic stress and silver nanoparticles, also found hundreds of differentially regulated proteins (Kamath et al., 2017; Liao et al., 2019).

Additionally, the samples from strains CCUG 51971 and CCUG 70744 exposed to no antibiotic were compared with the samples from strain CCUG 56489 (= PAO1) to determine differences in basal abundances. For strain CCUG 51971, a total of 2,573 proteins fulfilled the criteria for being included in the comparison (i.e., they were detected with >1 peptide, quantitated and shared >99% identity and 100% coverage with their corresponding homolog in PAO1). Of those, 335 (13%) and 423 (16%) presented higher and lower basal relative abundances, respectively (Supplementary Table 2). For strain CCUG 70744, 2,857 proteins fulfilled the inclusion criteria, of which 403 (14%) and 459 (16%) presented significantly higher and lower basal relative abundances, respectively (Supplementary Table 3). The fact that, in both strains, almost 30% of the proteins that were compared with strain PAO1 presented significantly higher or lower basal relative abundances could be expected, considering that they are different strains with a different genomic context, but establishes a base for future studies aiming to confirm them and determine the possible implications for antimicrobial resistance.

### 3.4. Differentially regulated β-lactamases

*Pseudomonas aeruginosa* harbors several naturally-occurring (Lodge et al., 1990; Girlich et al., 2004; Fajardo et al., 2014) and sometimes horizontally-acquired β-lactamases with various degrees of hydrolytic activity (Zhao and Hu, 2010). To elucidate involvements of β-lactamases in the response to carbapenems in the two carbapenem-resistant strains included in this study, we looked at the relative abundances of these enzymes under the different sub-MICs of meropenem (Table 2).

**Table 2:** β-lactamases encoded by *Pseudomonas aeruginosa* strains CCUG 51971 and CCUG 70744 and their relative abundances.

									8/2 vs. 0 μg/ml			128/4 vs. 0 μg/ml			256/8 vs. 0 μg/ml			0 µg/ml VS CCUG 56489 (= PAO1) 0 µg/ml		
Strain	β-lactamase	Ambler class	Subfamily	Origin	Accession	PAO1 ortholog	# Peptides	# PSMs	Fold change	*p*-value	Reg.	Fold change	*p*-value	Reg.	Fold change	*p*-value	Reg.	Fold change	*p*-value	Basal abundance
CCUG 51971	VIM-4	B1	VIM-1-like	Acquired	WP_032492008.1	-	6	38	1.02	0.81		−1.03	0.78		24.91	0.00	↑ Up			
	PDC-35	C	-	Natural	WP_003093423.1	PA4110	14	75	−1.33	0.11		−1.23	0.19		1.75	0.01	↑ Up			
	OXA-488	D	OXA-50-like	Natural	WP_003097028.1	PA5514	3	4	−1.38	0.35		1.18	0.70		−2.17	0.08		−1.20	0.61	
	OXA-35	D	OXA-10-like	Acquired	WP_063862720.1	-	8	85	−1.49	0.00		1.11	0.01		−1.67	0.00	↓ Down			
	-	B/C	PIB-1-like	Natural	WP_003097097.1	PA5542	3	4	n/q	-		n/q	-		n/q	-		n/q	-	
CCUG 70744	PDC-8	C	-	Natural	WP_003118203.1	PA4110	19	185	3.26	0.00	↑ Up	10.04	0.00	↑ Up	11.30	0.00	↑ Up	1.21	0.05	
	OXA-905	D	OXA-50-like	Natural	WP_003161096.1	PA5514	3	4	1.05	0.71		1.35	0.08		1.34	0.14		0.91	0.53	
	-	B/C	PIB-1-like	Natural	WP_003122311.1	PA5542	10	20	−1.15	0.00		1.09	0.04		−1.44	0.00		2.00	0.00	↑ Higher

n/q, not quantitated; reg., regulation.

For strain CCUG 51971, a previous study showed that a class 1 integron-encoded VIM-4 MBL was responsible for its high levels of carbapenem-resistance (Giske et al., 2003). The analysis of the complete genome sequence, using ICEfinder, indicates that VIM-4 is encoded in a putative ICE of 100 kb with a type IV secretion system. The quantitative proteomic analysis showed no significant changes in the relative abundance of VIM-4 in cultures containing 8 and 128 μg/ml, suggesting that the basal expression levels of *bla*_VIM-4_ are enough for the strain to thrive in these concentrations. However, at 256 μg/ml, VIM-4 was highly upregulated, with a fold change of 24.91, suggesting that the cells were struggling to cope with 256 μg/ml of meropenem.

Both strains encode a naturally-occurring and inducible AmpC-type Ambler class C β-lactamase (*Pseudomonas*-derived cephalosporinase, PDC). Strain CCUG 51971 encodes a PDC-35 β-lactamase, which was upregulated at 256 μg/ml. Strain CCUG 70744 encodes a PDC-8 β-lactamase, which was upregulated at all three sub-MICs (fold changes: 3.26, 10.04, and 11.30). Both PDC variants have an alanine residue at position 105, which has been previously associated with carbapenemase activity and reduced susceptibility to imipenem (Rodríguez-Martínez et al., 2009a). Thus, the upregulation of these PDCs with possible extended-spectrum activity might contribute to the carbapenem-resistance of these strains.

Each carbapenem-resistant strain also encodes a naturally-occurring OXA-50-like class D β-lactamase (OXA-488 and OXA-905, respectively). In both cases, the enzyme was detected and quantitated but remained stable in all conditions, which is in accordance with previous studies that failed to induce *bla*_OXA-50_, using β-lactams (Girlich et al., 2004; Zincke et al., 2016). However, like OXA-50, they may have weak carbapenem hydrolytic activity (Girlich et al., 2004; Zincke et al., 2016). In addition, strain CCUG 51971 also harbors a class 1 integron-located OXA-10-like restricted-spectrum oxacillinase (OXA-35), which appeared slightly downregulated at the highest sub-MIC (−1.67-fold) and which previously has been shown to be inhibited by imipenem (Aubert et al., 2001).

Finally, both carbapenem-resistant strains harbor a naturally-occurring PIB-1-like Zn^2+^-dependent imipenemase. In strain CCUG 51971, the protein was detected, but could not be quantitated, probably because it was produced at levels too low to measure. In strain CCUG 70744, the same protein was detected and quantitated, but not differentially regulated in any condition. However, the relative abundance of PIB-1 in strain CCUG 70744 was double than the one observed in strain PAO1, which has been previously suggested to contribute to carbapenem resistance (Fajardo et al., 2014).

### 3.5. Differentially regulated porins, efflux pumps and other transport-related proteins

In Gram-negative bacteria, β-lactam antibiotics need to cross the outer membrane *via* porins (i.e., water-filled protein channels) in order to reach the target PBPs, which are located on the surface of the cytoplasmatic membrane (Sauvage et al., 2008). By default, the outer membrane permeability of *P. aeruginosa* is 12–100-fold lower than that of *E. coli* (Yoshimura and Nikaido, 1982; Nikaido and Hancock, 1986), partly due to the lack of non-specific general diffusion porins and to narrower specific porins (Eren et al., 2012; Chevalier et al., 2017). Since porins can be involved in the import of antibiotics, we looked for porins that were differentially regulated when the carbapenem-resistant strains were exposed to sub-MICs of meropenem.

Firstly, we looked at the outer membrane porin OprD, which mainly serves for the transport of nutrients, such as basic amino acids, but also aids the intake of carbapenems and is considered a major resistance determinant (Li et al., 2012). In strain CCUG 51971, the genome sequence revealed an insertion causing a frameshift (1206insC; locus tag: FZE14_RS23605) and an altered 57 amino acids longer version of the protein. The quantitative proteomic analysis revealed only residual abundance ratios in all four conditions, suggesting that the protein is either not produced or produced at very low levels. In strain CCUG 70744, OprD was produced, but downregulated at the two highest sub-MICs, showing the largest fold change at the highest sub-MIC (Table 3). Additionally, inactivation of OprD also has been associated with enhanced fitness and virulence (Skurnik et al., 2013), indicating that this could also led to increased pathogenicity.

**Table 3:** Differentially regulated porins of *Pseudomonas aeruginosa* CCUG 51971 and CCUG 70744.

						8/2 vs. 0 μg/ml			128/4 vs. 0 μg/ml			256/8 vs. 0 μg/ml			0 µg/ml VS CCUG 56489 (= PAO1) 0 µg/ml		
Strain	Product	RefSeq accession	PAO1 ortholog	# Peptides	# PSMs	Fold change	*p*-value	Reg.	Fold change	*p*-value	Reg.	Fold change	*p*-value	Reg.	Fold change	*p*-value	Basal abundance
CCUG 51971	OpdC (OccD2)	WP_003083884.1	PA0162	9	27	−1.01	0.75		−1.14	0.01		−1.19	0.00		−2.37	0.00	↓ Lower
	OprE (OccK8)	WP_003084265.1	PA0291	17	71	1.00	0.91		−1.19	0.10		−1.09	0.39		−1.05	0.46	
	OpdH (OccK5)	WP_023098812.1	PA0755	7	20	1.20	0.15		−1.21	0.13		1.43	0.01		−1.03	0.72	
	OprD (OccD1)	-	PA0958	0	0												
	**OprH**	**WP_003082431.1**	**PA1178**	9	210	−1.42	0.13		−1.09	0.78		−4.15	0.00	↓ Down	−1.18	0.58	
	AprF	WP_003086727.1	PA1248	7	12	−2.76	0.00	↓ Down	−1.02	0.92		−8.37	0.00	↓ Down	1.55	0.03	↑ Higher
	**OprF**	**WP_003087843.1**	**PA1777**	14	247	2.87	0.00	↑ Up	2.36	0.00	↑ Up	1.11	0.64		−1.99	0.00	↓ Lower
	OpmL	WP_003102743.1	PA1875	4	6	1.87	0.00	↑ Up	−1.25	0.05		−3.07	0.00	↓ Down	−1.48	0.01	
	**OpdO (OccK3)**	**WP_023464697.1**	**PA2113**	14	37	1.28	0.01		−1.11	0.07		−9.33	0.00	↓ Down	−1.36	0.00	
	OprB2 (OpbA)	WP_031640100.1	PA2291	0	0												
	OpdT (OprD3)	WP_003109544.1	PA2505	0	0												
	OprQ (OccD6)	WP_003099122.1	PA2760	13	101	1.04	0.32		−1.22	0.00		−1.46	0.00				
	**OpdQ (OccK6)**	**WP_003091257.1**	**PA3038**	13	47	1.05	0.30		−1.16	0.02		−5.22	0.00	↓ Down	−1.51	0.00	↓ Lower
	**OprB**	**WP_003116413.1**	**PA3186**	15	92	1.25	0.00		−1.09	0.10		−7.54	0.00	↓ Down	1.01	0.89	
	**OprO**	**WP_003104423.1**	**PA3280**	16	43	1.19	0.03		−1.13	0.05		−5.06	0.00	↓ Down	−1.81	0.00	↓ Lower
	OprC	WP_003119294.1	PA3790	22	85	−1.16	0.15		−1.09	0.38		−2.21	0.00	↓ Down	1.11	0.26	
	**OprG**	**WP_003093337.1**	**PA4067**	6	42	−1.33	0.00		−2.23	0.00	↓ Down	−6.22	0.00	↓ Down	2.36	0.00	↑ Higher
	OpdL (OccK4)	WP_003105682.1	PA4137	1	2	1.23	0.16		−1.36	0.12		−4.39	0.01				
	**OpdP (OccD3)**	**WP_023465243.1**	**PA4501**	5	14	−1.05	0.57		−1.19	0.04		−5.65	0.00	↓ Down			
	**OpmH**	**WP_110183992.1**	**PA4974**	27	99	−1.10	0.07		−1.22	0.00		−2.70	0.00	↓ Down	1.35	0.00	
CCUG 70744	OpdC (OccD2)	WP_003112647.1	PA0162	9	21	−1.34	0.00		−2.58	0.00	↓ Down	−3.12	0.00	↓ Down	−1.26	0.00		
	OprE (OccK8)	WP_003084265.1	PA0291	15	70	1.00	0.90		−1.31	0.00		−1.61	0.00	↓ Down	0.95	0.28		
	OpdH (OccK5)	WP_023464305.1	PA0755	8	16	1.12	0.00		−1.11	0.00		−1.85	0.00	↓ Down	1.48	0.00		
	OprD (OccD1)	WP_110183965.1	PA0958	11	95	−1.10	0.07		−2.14	0.00	↓ Down	−4.51	0.00	↓ Down				
	**OprH**	**WP_003082431.1**	**PA1178**	10	298	1.04	0.21		−1.54	0.00	↓ Down	−1.93	0.00	↓ Down	−1.18	0.00		
	AprF	WP_003086727.1	PA1248	1	1													
	**OprF**	**WP_003087843.1**	**PA1777**	12	208	1.02	0.81		−1.56	0.00	↓ Down	−1.57	0.00	↓ Down	−1.85	0.00	↓ Lower	
	OpmL	WP_003102743.1	PA1875	2	2	n/q	-		n/q	-		n/q	-		n/q	-		
	**OpdO (OccK3)**	**WP_023464697.1**	**PA2113**	17	53	−1.01	0.61		−1.68	0.00	↓ Down	−2.58	0.00	↓ Down	1.83	0.00	↑ Higher	
	OprB2 (OpbA)	WP_031640100.1	PA2291	16	109	−1.15	0.16		−2.10	0.00	↓ Down	−3.20	0.00	↓ Down	41.38	0.01	↑ Higher	
	OpdT (OprD3)	WP_003109544.1	PA2505	2	3	−1.09	0.54		3.35	0.00	↑ Up	2.30	0.00	↑ Up	1.56	0.12		
	OprQ (OccD6)	WP_003099122.1	PA2760	15	90	−1.12	0.00		−1.55	0.00	↓ Down	−2.12	0.00	↓ Down	−1.52	0.00	↓ Lower	
	**OpdQ (OccK6)**	**WP_003091257.1**	**PA3038**	14	54	−1.19	0.00		−1.87	0.00	↓ Down	−3.15	0.00	↓ Down	1.38	0.00		
	**OprB**	**WP_003116413.1**	**PA3186**	17	124	−1.13	0.01		−2.04	0.00	↓ Down	−1.51	0.00	↓ Down	1.26	0.00		
	**OprO**	**WP_003104423.1**	**PA3280**	16	50	−1.29	0.00		−2.05	0.00	↓ Down	−2.32	0.00	↓ Down	1.76	0.00	↑ Higher	
	OprC	WP_003119294.1	PA3790	24	104	1.02	0.52		−1.11	0.01		−1.32	0.00		1.43	0.00		
	**OprG**	**WP_003093337.1**	**PA4067**	7	47	−1.13	0.00		−2.09	0.00	↓ Down	−3.38	0.00	↓ Down	3.90	0.00	↑ Higher	
	OpdL (OccK4)	WP_003105682.1	PA4137	2	4	1.01	0.74		−1.67	0.00	↓ Down	−6.77	0.00	↓ Down	4.74	0.00	↑ Higher	
	**OpdP (OccD3)**	**WP_023465243.1**	**PA4501**	5	11	−1.09	0.39		−1.03	0.81		−1.60	0.05	↓ Down				
	**OpmH**	**WP_110183992.1**	**PA4974**	23	84	−1.08	0.00		−1.34	0.00		−1.90	0.00	↓ Down	−1.07	0.01	

Orthologous proteins differentially regulated in both strains are in bold and shaded. n/q, not quantitated; reg., regulation.

The genome sequence of strain CCUG 51971 also revealed that the gene encoding OpdD, another porin which can facilitate uptake of meropenem (Isabella et al., 2015), was frameshifted by a deletion (213del; locus tag: FZE14_RS22945) that generates a premature stop codon. In strain CCUG 70744, OpdD seemed functional in the genome sequence but was not detected by the proteomic analysis, which could be due to very low abundances.

In both carbapenem-resistant strains, the majority of porins that were deemed differentially regulated when exposed to sub-MICs of meropenem, were downregulated. In strain CCUG 51971, 11 porins were downregulated at the highest sub-MIC (Table 3). Of those, OprG was also downregulated at the middle sub-MIC and AprF at the lowest. Strikingly, OprF appeared upregulated at 8 and 128 μg/ml and OpmL upregulated at 8 μg/ml, but none of them at 256 μg/ml. In strain CCUG 70744, 16 porins were downregulated at the highest sub-MIC, four of them only in that condition and 11 both at the highest and the middle sub-MICs. Interestingly, OpdT was upregulated at 4 and 8 μg/ml. Of all these porins, eight were downregulated in both strains (OprH, OpdP, OpdO, OpdQ, OprB, OprO, OprG, and OpmH), suggesting that they might be conserved responses to meropenem in *P. aeruginosa*. Of these, OpdP has been demonstrated to be involved in meropenem uptake (Isabella et al., 2015).

Additionally, *P. aeruginosa* harbors a large arsenal of efflux pumps, some of which have been associated with natural and acquired antibiotic resistance caused by extrusion of antibiotics to the outside of the cells (Li and Plésiat, 2016). Hence, we searched for differentially regulated components of the characterized drug efflux pumps listed by Li and Plésiat (2016) (Table 4).

**Table 4:** Characterized drug efflux pumps of *Pseudomonas aeruginosa* CCUG 51971 and CCUG 70744 with differentially regulated proteins.

								8/2 vs. 0 μg/ml			128/4 vs. 0 μg/ml			256/8 vs. 0 μg/ml			0 µg/ml VS CCUG 56489 (= PAO1) 0 µg/ml		
Strain	Transporter family	Efflux pump	Product	RefSeq accession	PAO1 ortholog	# Peptides	# PSMs	Fold change	*p*-value	Reg.	Fold change	*p*-value	Reg.	Fold change	*p*-value	Reg.	Fold change	*p*-value	Basal abundance
CCUG 51971	RND	MexCD-OprJ	MexC	WP_023657821.1	PA4599	12	30	−1.03	0.73		−1.07	0.48		1.98	0.00	↑ Up			
			MexD	WP_003094838.1	PA4598	13	21	1.10	0.14		1.12	0.22		1.93	0.00	↑ Up	1.28	0.00	
			OprJ	WP_004352729.1	PA4597	3	7	−1.03	0.66		−1.15	0.08		1.60	0.00	↑ Up	3.94	0.01	↑ Higher
		MexEF-OprN	MexE	WP_003089831.1	PA2493	4	6	−1.40	0.01		−1.19	0.15		2.76	0.01	↑ Up	−1.07	0.63	
			MexF	WP_003089834.1	PA2494	2	4	n/q	-		n/q	-		n/q	-				
			OprN	WP_003089836.1	PA2495	0	0												
		MexGHI-OpmD	MexG	WP_003093607.1	PA4205	2	6	1.60	0.24		−1.30	0.78		1.15	0.47		−17.77	0.00	↓ Lower
			MexH	WP_003093609.1	PA4206	13	41	1.37	0.04		−1.11	0.45		1.05	0.79		−24.68	0.00	↓ Lower
			MexI	WP_003093610.1	PA4207	22	50	1.33	0.14		1.02	0.83		1.67	0.04	↑ Up	−35.62	0.00	↓ Lower
			OpmD	WP_003093612.1	PA4208	15	36	1.19	0.07		−1.16	0.03		1.47	0.01		−30.31	0.00	↓ Lower
		MexJK-OprM/OpmH	**MexJ**	**WP_003113855.1**	**PA3677**	10	25	−1.27	0.00		−1.20	0.00		−3.56	0.00	↓ Down	1.47	0.09	
			**MexK**	**WP_003092464.1**	**PA3676**	18	43	1.02	0.40		1.07	0.31		−4.18	0.00	↓ Down	1.41	0.00	
			OprM	WP_003084633.1	PA0427	15	44	−1.53	0.13		−1.47	0.16		−1.39	0.23				
			**OpmH**	**WP_110183992.1**	**PA4974**	27	99	−1.10	0.07		−1.22	0.00		−2.70	0.00	↓ Down	1.35	0.00	
		MexVW-OprM	MexV	WP_023128433.1	PA4374	7	20	−1.15	0.01		−1.21	0.00		1.24	0.00		1.07	0.20	
			MexW	WP_003141256.1	PA4375	10	18	−1.08	0.38		−1.08	0.35		1.35	0.01		−1.17	0.08	
			OprM	WP_003084633.1	PA0427	15	44	−1.53	0.13		−1.47	0.16		−1.39	0.23				
		MexXY-OprM	MexX	WP_031636852.1	PA2019	17	63	1.12	0.15		−1.14	0.01		2.04	0.00	↑ Up	5.75	0.00	↑ Higher
			MexY	WP_003088621.1	PA2018	24	111	1.22	0.02		1.07	0.34		1.50	0.00	↑ Up	4.16	0.01	↑ Higher
			OprM	WP_003084633.1	PA0427	15	44	−1.53	0.13		−1.47	0.16		−1.39	0.23				
		TriABC-OprM/OprH	**TriA**	**WP_003118583.1**	**PA0156**	15	42	1.00	0.95		−1.26	0.00		−3.00	0.00	↓ Down			
			TriB	WP_003161029.1	PA0157	17	41	1.01	0.76		−1.24	0.00		−2.63	0.00	↓ Down	1.84	0.00	↑ Higher
			**TriC**	**WP_003102199.1**	**PA0158**	21	55	1.21	0.00		1.03	0.71		−2.72	0.00	↓ Down	1.15	0.00	
			OprM	WP_003084633.1	PA0427	15	44	−1.53	0.13		−1.47	0.16		−1.39	0.23				
			**OprH**	**WP_003082431.1**	**PA1178**	9	210	−1.09	0.78		−1.42	0.13		−4.15	0.00	↓ Down	−1.18	0.58		
	ABC	PA1874-1877	PA1874	WP_031637117.1	PA1874	4	15	2.26	0.00	↑ Up	−1.15	0.07		−6.43	0.00	↓ Down			
			PA1875/OpmL	WP_003102743.1	PA1875	4	6	1.87	0.00	↑ Up	−1.25	0.05		−3.07	0.00	↓ Down	−1.48	0.01	
			PA1876	WP_003088105.1	PA1876	3	3	n/q	-		n/q	-		n/q	-				
			PA1877	WP_003088106.1	PA1877	0	0												
		Ttg2ABCDE	Ttg2E	WP_003094335.1	PA4452	3	15	−1.19	0.02		−1.30	0.00		1.35	0.00		1.24	0.00	
			Ttg2D	WP_003094337.1	PA4453	11	57	−1.20	0.02		1.43	0.00		2.36	0.00	↑ Up	−2.23	0.00	↓ Lower
			Ttg2C	WP_003094339.1	PA4454	5	18	1.28	0.88		−1.08	0.68		1.87	0.01	↑ Up	−1.22	0.17	
			Ttg2B	WP_003094341.1	PA4455	3	19	−1.03	0.66		−1.23	0.01		1.42	0.00		−1.06	0.30	
			Ttg2A	WP_003094343.1	PA4456	11	46	−1.01	0.81		−1.11	0.04		1.62	0.00	↑ Up	1.24	0.00			
		PvdRT-OpmQ	PvdR	WP_003089547.1	PA2389	1	1	-	-		-	-		-	-						
			PvdT	WP_003089549.1	PA2390	5	6	n/q	-		n/q	-		n/q	-						
			OpmQ	WP_073660165.1	PA2391	7	11	1.09	0.08		1.39	0.00		−1.80	0.00	↓ Down	−1.71	0.00	↓ Lower		
		PA1113	PA1113	WP_003086478.1	PA1113	9	13	−1.26	0.00		−1.12	0.07		−2.18	0.01	↓ Down	−1.02	0.80	
CCUG 70744	RND	MexCD-OprJ	MexC	WP_004352726.1	PA4599	0	0												
			MexD	WP_003103150.1	PA4598	4	7	n/q	-		n/q	-		n/q	-				
			OprJ	WP_004352729.1	PA4597	0	0												
		MexEF-OprN	MexE	WP_003103549.1	PA2493	1	1												
			MexF	WP_003089834.1	PA2494	2	5	n/q	-		n/q	-		n/q	-				
			OprN	WP_003113303.1	PA2495	0	0												
		MexGHI-OpmD	MexG	WP_003093607.1	PA4205	2	8	n/q	-		n/q	-		n/q	-				
			MexH	WP_023835860.1	PA4206	12	34	1.02	0.91		1.17	0.70		−1.29	0.50		−21.97	0.00	↓ Lower
			MexI	WP_003114727.1	PA4207	20	41	1.02	0.79		1.01	0.87		1.01	0.81		−30.07	0.00	↓ Lower
			OpmD	WP_023465199.1	PA4208	13	33	1.13	0.31		1.07	0.54		1.00	0.98		−23.40	0.00	↓ Lower
		MexJK-OprM/OpmH	**MexJ**	**WP_003113855.1**	**PA3677**	12	29	−1.29	0.00		−1.50	0.00	↓ Down	−1.33	0.00		1.20	0.01	
			**MexK**	**WP_003092464.1**	**PA3676**	20	43	−1.16	0.00		−1.43	0.00		−1.51	0.00	↓ Down	−1.11	0.02	
			OprM	WP_003084633.1	PA0427	18	52	−1.07	0.30		1.29	0.00		1.62	0.10				
			**OprH**	**WP_003082431.1**	**PA1178**	10	298	1.04	0.21		−1.54	0.00	↓ Down	−1.93	0.00	↓ Down			
		MexVW-OprM	MexV	WP_023128433.1	PA4374	6	16	−1.05	0.05		−1.37	0.00		−1.59	0.00	↓ Down	−1.61	0.00	↓ Lower
			MexW	WP_003141256.1	PA4375	10	16	−1.06	0.09		−1.19	0.00		−1.14	0.04		−1.56	0.00	↓ Lower
			OprM	WP_003084633.1	PA0427	18	52	−1.07	0.30		1.29	0.00		1.62	0.10		−2.84	0.00	↓ Lower
		MexXY-OprM	MexX	WP_031636852.1	PA2019	16	65	−1.01	0.65		−1.30	0.00		−1.28	0.00		7.93	0.00	↑ Higher
			MexY	WP_003088621.1	PA2018	22	98	−1.03	0.19		−1.29	0.00		−1.30	0.00		5.12	0.00	↑ Higher
			OprM	WP_003084633.1	PA0427	18	52	−1.07	0.30		1.29	0.00		1.62	0.10						
		TriABC-OprM/OprH	**TriA**	**WP_003118583.1**	**PA0156**	13	24	1.04	0.08		−1.14	0.01		−1.61	0.00	↓ Down	−1.48	0.00			
			TriB	WP_003161029.1	PA0157	15	30	1.05	0.17		−1.03	0.50		−1.32	0.00		−1.76	0.00	↓ Lower		
			**TriC**	**WP_003102199.1**	**PA0158**	21	42	1.04	0.09		−1.10	0.01		−1.57	0.00	↓ Down	−2.26	0.00	↓ Lower		
			OprM	WP_003084633.1	PA0427	18	52	−1.07	0.30		1.29	0.00		1.62	0.10						
			**OprH**	**WP_003082431.1**	**PA1178**	10	298	1.04	0.21		−1.54	0.00	↓ Down	−1.93	0.00	↓ Down	−1.18	0.00		
	ABC	PA1874-1877	PA1874	WP_031637117.1	PA1874	1	2	1.77	0.07		1.73	0.33		1.44	0.47						
			PA1875/OpmL	WP_003102743.1	PA1875	2	2	n/q	-		n/q	-		n/q	-						
			PA1876	WP_003114950.1	PA1876	0	0														
			PA1877	WP_003102741.1	PA1877	0	0														
		Ttg2ABCDE	Ttg2E	WP_003094335.1	PA4452	3	20	1.05	0.10		1.18	0.00		1.30	0.00		0.90	0.35			
			Ttg2D	WP_003094337.1	PA4453	11	65	1.02	0.54		1.29	0.00		1.34	0.00		−1.29	0.00			
			Ttg2C	WP_003094339.1	PA4454	6	25	1.03	0.76		−1.23	0.12		−1.01	0.81		1.02	0.74			
			Ttg2B	WP_003094341.1	PA4455	4	21	1.05	0.06		−1.07	0.01		−1.02	0.36		1.13	0.01			
			Ttg2A	WP_110183972.1	PA4456	10	43	1.05	0.02		−1.12	0.00		−1.06	0.02		1.33	0.00			
		PvdRT-OpmQ	PvdR	WP_003114507.1	PA2389	1	1	n/q	-		n/q	-		n/q	-						
			PvdT	WP_003089549.1	PA2390	1	2	n/q	-		n/q	-		n/q	-						
			OpmQ	WP_003120134.1	PA2391	3	4	1.08	0.62		1.30	0.21		1.26	0.48		−3.09	0.02	↓ Lower		
		PA1113	PA1113	WP_003145485.1	PA1113	6	12	1.02	0.68		−1.08	0.05		−1.21	0.02		1.12	0.01	

Orthologous proteins differentially regulated in both strains are in bold and shaded. n/q, not quantitated; reg., regulation.

In strain CCUG 51971, several efflux pumps of the resistance-nodulation-cell division (RND) family were upregulated at 256 μg/ml. Among those, were included the three components of the MexCD-OprJ efflux pump, which can extrude meropenem (Masuda et al., 2000). The protein MexE of the MexEF-OprN efflux pump was also upregulated, although the MexF and OprN components were not quantitated nor detected, respectively. The MeXY components of the MexXY-OprM pump, which is also able to pump out meropenem (Masuda et al., 2000), also were upregulated. Moreover, compared to strain PAO1, the basal relative abundances of the MexXY components were already 4–6-fold higher. Finally, the MexI component of the MexGHI-OpmD efflux pump was also upregulated, although not the other components. Additionally, the proteins Ttg2A, Ttg2C and Ttg2D of the Ttg2 ATP-binding cassette (ABC) transporter, also were upregulated. This transporter is associated with membrane-phospholipid homeostasis, outer membrane permeability and has been previously associated to low level resistance to various relatively hydrophobic antibiotics (Yero et al., 2021).

In contrast, all components of the MexJK-OpmH and TriABC-OprH RND efflux pumps were downregulated at 256 μg/ml, as well as the outer membrane protein of the PvdRT-OpmQ ABC transporter. Interestingly, the two components of the ABC transporter encoded by the *P. aeruginosa* locus tags PA1874-1877 that were quantitated, appeared downregulated at 256 μg/ml but upregulated at 8 μg/ml.

In strain CCUG 70744, fewer efflux pumps had differentially regulated protein subunits. Like in strain CCG 51971, the RND efflux pumps MexJK-OprH and TriABC-OprH had downregulated components (MexJ at 4 μg/ml; MexK, TriA and TriC at 8 μg/ml, and OprH at 4 and 8 μg/ml). Additionally, the protein MexV of the MexVW-OprM efflux pump was slightly downregulated. The MexAB-OprM efflux pump has been shown to extrude meropenem (Masuda et al., 2000); however, this system was not differentially regulated in any of the strains and did not present higher protein abundances compared to strain PAO1, although the three proteins of the system were detected in both cases with more than 10 peptides. The MexXY-OprM efflux pump was not differentially regulated with meropenem exposure. However, when compared with strain PAO1, the MeXY components had a significantly higher relative abundance (MexX: 7.93-fold; MexY: 5.12-fold), as observed also in strain CCUG 51971. This confirms the prediction made by Johnning et al., who reported an insertion impairing the function of the repressor *mexZ* in the genome of strain CCUG 70744 (Johnning et al., 2018), which has been previously linked to overexpression of MeXY (Vogne et al., 2004), which may contribute to the low meropenem susceptibility of the strain (Yero et al., 2021).

Since lowering the outer membrane permeability and increasing efflux pumps expression are major mechanisms of resistance in *P. aeruginosa*, we searched for additional transmembrane transport-related proteins that were differentially regulated. Using several selected GO terms, 55 and 22 proteins were found to be potentially associated with transmembrane transport and differentially regulated in strains CCUG 51971 and CCUG 70744, respectively (Table 5). Most of those proteins were downregulated (40 of 55 and 20 of 22) and 14 were differentially regulated in both strains, suggesting that they might be involved in conserved responses to meropenem.

**Table 5:** Additional differentially regulated proteins related with transmembrane transport.

							8/2 vs. 0 μg/ml			128/4 vs. 0 μg/ml			256/8 vs. 0 μg/ml			0 µg/ml VS CCUG 56489 (= PAO1) 0 µg/ml		
Strain	Accession	PAO1 ortholog	Product	Product description (*Pseudomonas* genome database)	# Peptides	# PSMs	Fold change	*p*-value	Reg.	Fold change	*p*-value	Reg.	Fold change	*p*-value	Reg.	Fold change	*p*-value	Basal abundance
CCUG 51971	**WP_003089559.1**	**-**	-	-	3	6	1.21	0.39		1.84	0.08		−11.02	0.03	↓ Down				
	WP_003136959.1	PA0073	TagT1	TagT1	6	12	1.01	0.88		−1.04	0.62		1.76	0.00	↑ Up	3.89	0.00	↑ Higher	
	WP_003083784.1	PA0129	BauD	Amino acid permease	2	4	1.01	0.85		1.41	0.01		−2.27	0.00	↓ Down	−2.00	0.00	↓ Lower	
	**WP_003121722.1**	**PA0303**	SpuG/PotH	Polyamine transport protein PotH	3	9	−1.09	0.38		−1.08	0.41		−1.76	0.00	↓ Down	−1.10	0.26		
	WP_014604087.1	PA0605	AgtC	AgtC	6	15	−1.09	0.20		−1.09	0.24		−2.59	0.00	↓ Down	1.12	0.08		
	WP_003085613.1	PA0789	-	Probable amino acid permease	5	13	1.02	0.91		1.50	0.00	↑ Up	4.69	0.00	↑ Up	−1.80	0.00	↓ Lower	
	WP_003085934.1	PA0889	AotQ	Arginine/ornithine transport protein AotQ	3	9	1.09	0.58		1.24	0.15		2.04	0.00	↑ Up	1.00	0.98		
	WP_003085936.1	PA0890	AotM	arginine/ornithine transport protein AotM	2	6	−1.15	0.42		−1.08	0.70		1.64	0.02	↑ Up	−1.09	0.47		
	WP_003086854.1	PA1316	-	Probable major facilitator superfamily (MFS) transporter	3	5	1.38	0.22		−1.29	0.12		−2.23	0.00	↓ Down	44.40	0.01	↑ Higher	
	WP_003082767.1	PA1341	AatQ	AatQ	3	10	−1.14	0.01		−1.08	0.04		−2.55	0.00	↓ Down	1.44	0.00		
	**WP_003086995.1**	**PA1408**	-	Hypothetical protein	13	32	−1.05	0.50		−1.02	0.83		−4.55	0.00	↓ Down	1.20	0.08		
	WP_003087942.1	PA1808	NppC/OppD	NppC	3	7	1.02	0.65		1.14	0.03		−1.54	0.00	↓ Down	−1.20	0.03		
	WP_003087943.1	PA1809	NppB/OppC	NppB	7	17	1.00	0.96		1.10	0.28		−1.61	0.01	↓ Down	1.18	0.14		
	**WP_003087965.1**	**PA1819**	YjdE	Probable amino acid permease	3	7	−1.09	0.06		−1.25	0.00		−4.78	0.00	↓ Down	−1.35	0.00		
	**WP_003088303.1**	**PA1948**	RbsC	Membrane protein component of ABC ribose transporter	3	14	−1.05	0.43		1.11	0.08		−5.66	0.00	↓ Down	1.04	0.52		
	WP_003088580.1	PA2006	-	Probable major facilitator superfamily (MFS) transporter	3	6	−1.04	0.56		−1.02	0.76		−6.45	0.00	↓ Down	−1.06	0.55		
	**WP_003089254.1**	**PA2252**	-	Probable AGCS sodium/alanine/glycine symporter	2	5	−1.04	0.66		1.23	0.02		3.57	0.00	↑ Up	−2.73	0.00	↓ Lower	
	WP_003089429.1	PA2328	-	Hypothetical protein	3	6	−1.15	0.08		−2.72	0.00	↓ Down	−3.33	0.00	↓ Down	−1.93	0.00	↓ Lower	
	**WP_003089954.1**	**PA2533**	-	Probable sodium:alanine symporter	2	8	1.01	0.90		1.07	0.78		−2.17	0.01	↓ Down	1.06	0.68		
	**WP_003090842.1**	**PA2777**	YfdC	Conserved hypothetical protein	3	7	−1.13	0.28		−1.76	0.00	↓ Down	−3.81	0.00	↓ Down				
	WP_003091187.1	PA3000	AroP1	Aromatic amino acid transport protein AroP1	2	4	1.00	0.90		1.34	0.02		2.11	0.00	↑ Up	−1.27	0.04		
	WP_003091470.1	PA3188	GltG	Probable permease of ABC sugar transporter	2	7	1.20	0.03		1.23	0.04		−3.84	0.01	↓ Down	1.26	0.01		
	WP_003091471.1	PA3189	GltF	Probable permease of ABC sugar transporter	4	11	−1.09	0.59		1.25	0.17		−10.82	0.00	↓ Down	1.30	0.09		
	WP_003091539.1	PA3228	-	Probable ATP-binding/permease fusion ABC transporter	13	35	−1.07	0.02		−1.31	0.00		−1.99	0.00	↓ Down	1.57	0.01	↑ Higher	
	**WP_021265067.1**	**PA3236**	BetX	BetX/betaine-specific binding protein	5	15	−1.41	0.00		−1.71	0.00	↓ Down	−3.32	0.00	↓ Down	−2.81	0.00	↓ Lower	
	WP_003091590.1	PA3254	-	Probable ATP-binding component of ABC transporter	10	25	−1.05	0.11		−1.07	0.07		−7.28	0.00	↓ Down	2.09	0.00	↑ Higher	
	**WP_003091605.1**	**PA3267**	-	Hypothetical protein	6	15	1.08	0.54		1.12	0.02		1.74	0.00	↑ Up	1.06	0.20		
	WP_003091654.1	PA3304	-	Conserved hypothetical protein	6	13	−1.17	0.02		−1.27	0.00		−1.96	0.00	↓ Down	−1.26	0.01		
	WP_003091795.1	PA3383	PhnD	Binding protein component of ABC phosphonate transporter	17	88	1.32	0.00		−1.33	0.00		−9.05	0.00	↓ Down	−1.23	0.01		
	WP_020750987.1	PA3408	HasR	Heme uptake outer membrane receptor HasR precursor	3	6	−1.04	0.71		−1.39	0.02		−3.69	0.00	↓ Down	3.04	0.00	↑ Higher	
	**WP_003092370.1**	**PA3641**	-	Probable amino acid permease	4	19	−1.12	0.02		−1.07	0.11		1.80	0.00	↑ Up	−1.44	0.00		
	**WP_003092547.1**	**PA3709**	-	Probable major facilitator superfamily (MFS) transporter	6	22	1.14	0.11		1.43	0.00		−5.54	0.00	↓ Down	4.76	0.00	↑ Higher	
	WP_003092694.1	PA3766	-	Probable aromatic amino acid transporter	3	5	1.03	0.79		1.26	0.23		3.67	0.00	↑ Up	−1.24	0.35		
	WP_003114310.1	PA3934	-	Conserved hypothetical protein	4	11	1.03	0.48		−1.60	0.00	↓ Down	−1.75	0.01	↓ Down	1.38	0.00		
	WP_014604082.1	PA4222	PchI	Probable ATP-binding component of ABC transporter	19	55	−1.26	0.02		−1.68	0.00	↓ Down	−8.72	0.00	↓ Down				
	WP_023094503.1	PA4223	PchH	Probable ATP-binding component of ABC transporter	14	41	−1.23	0.00		−1.57	0.00	↓ Down	−11.90	0.00	↓ Down	7.45	0.00	↑ Higher	
	WP_003094425.1	PA4496	DppA1	Probable binding protein component of ABC transporter	13	31	1.18	0.00		−1.37	0.00		−6.69	0.00	↓ Down	−1.68	0.00	↓ Lower	
	WP_003094427.1	PA4497	DppA2	Probable binding protein component of ABC transporter	7	18	1.19	0.01		−1.51	0.00	↓ Down	−7.98	0.00	↓ Down				
	WP_034085213.1	PA4500	DppA3	Probable binding protein component of ABC transporter	8	20	1.28	0.15		1.01	0.85		1.54	0.04	↑ Up	−16.95	0.00	↓ Lower	
	WP_003094445.1	PA4502	DppA4	Probable binding protein component of ABC transporter	10	20	1.20	0.13		−1.45	0.03		−2.80	0.00	↓ Down	−1.69	0.01	↓ Lower	
	WP_003094448.1	PA4504	DppC	Dipeptide ABC transporter permease DppC	3	8	−1.10	0.60		−1.02	0.99		−1.68	0.02	↓ Down				
	WP_003094894.1	PA4616	-	Probable c4-dicarboxylate-binding protein	3	4	1.45	0.03		−1.14	0.37		5.36	0.00	↑ Up	−5.55	0.00	↓ Lower	
	WP_003094922.1	PA4628	LysP	lysine-specific permease	4	9	1.01	0.93		1.22	0.07		2.00	0.00	↑ Up	−1.22	0.03		
	WP_003121056.1	PA4688	HitB	iron (III)-transport system permease HitB	2	5	−1.20	0.18		−1.21	0.15		−1.71	0.01	↓ Down	−1.14	0.24		
	**WP_003095102.1**	**PA4710**	PhuR	Heme/Hemoglobin uptake outer membrane receptor PhuR precursor	21	61	−1.17	0.06		−1.32	0.01		−15.84	0.00	↓ Down	2.42	0.00	↑ Higher	
	WP_003095519.1	PA4887	-	Probable major facilitator superfamily (MFS) transporter	3	7	1.21	0.01		−1.73	0.01	↓ Down	2.68	0.00	↑ Up	1.05	0.49		
	**WP_003095599.1**	**PA4925**	-	Conserved hypothetical protein	3	15	−1.33	0.00		−1.03	0.34		−17.41	0.00	↓ Down	2.08	0.00	↑ Higher	
	WP_003095749.1	PA4997	MsbA	Transport protein MsbA	15	40	−1.04	0.43		1.00	1.00		1.55	0.00	↑ Up	1.26	0.00		
	WP_003095966.1	PA5103	PuuR	PuuR	6	11	1.20	0.00		−1.82	0.00	↓ Down	−3.51	0.00	↓ Down	−1.03	0.03		
	WP_003096160.1	PA5154	-	Probable permease of ABC transporter	4	11	−1.16	0.02		1.02	0.58		−2.10	0.02	↓ Down	−1.10	0.06		
	WP_003110670.1	PA5231	YhiH	Probable ATP-binding/permease fusion ABC transporter	9	18	1.03	0.59		−1.17	0.02		−1.79	0.00	↓ Down	1.12	0.03		
	WP_003096411.1	PA5268	CorA	Magnesium/cobalt transport protein	5	18	−1.17	0.00		1.01	0.66		−1.63	0.00	↓ Down	1.05	0.19		
	WP_003096557.1	PA5317	DppA5	Probable binding protein component of ABC dipeptide transporter	13	35	1.02	0.74		−1.62	0.00	↓ Down	−1.98	0.00	↓ Down	1.70	0.00	↑ Higher	
	WP_003123734.1	PA5368	PstC	Membrane protein component of ABC phosphate transporter	13	59	−1.08	0.01		1.48	0.00		−3.84	0.00	↓ Down	1.19	0.02		
	WP_003097074.1	PA5530	-	C5-dicarboxylate transporter	2	6	1.04	0.61		−1.06	0.53		5.53	0.00	↑ Up	2.58	0.00	↑ Higher
CCUG 70744	**WP_031636928.1**	**-**	-	-	10	20	−1.05	0.31		−1.48	0.00		−1.96	0.00	↓ Down				
	WP_003101889.1	PA0229	PcaT/KgtP	Dicarboxylic acid transporter PcaT	3	5	−1.07	0.40		−1.13	0.15		−1.63	0.00	↓ Down	−6.59	0.00	↓ Lower	
	**WP_003121722.1**	**PA0303**	SpuG/PotH	Polyamine transport protein PotH	3	7	−1.02	0.54		−1.30	0.01		−1.55	0.00	↓ Down	1.48	0.00		
	**WP_003162347.1**	**PA1408**	-	Hypothetical protein	12	30	1.08	0.09		1.00	0.96		−1.50	0.00	↓ Down	−1.57	0.00	↓ Lower	
	WP_003087830.1	PA1775	CmpX	Conserved cytoplasmic membrane protein, CmpX protein	6	22	1.02	0.19		−1.29	0.00		−1.55	0.00	↓ Down	−1.48	0.00		
	**WP_023464637.1**	**PA1819**	YjdE	Probable amino acid permease	4	6	1.00	0.90		−1.16	0.04		−1.72	0.00	↓ Down	1.18	0.02		
	**WP_003088303.1**	**PA1948**	RbsC	Membrane protein component of ABC ribose transporter	3	14	1.10	0.19		1.10	0.22		−1.81	0.00	↓ Down	1.66	0.00	↑ Higher	
	**WP_023464954.1**	**PA2252**	-	Probable AGCS sodium/alanine/glycine symporter	3	5	−1.08	0.40		−1.07	0.40		1.88	0.00	↑ Up	0.99	0.84		
	WP_003116900.1	PA2431	-	Hypothetical protein	2	4	1.10	0.05		−1.13	0.15		−2.40	0.00	↓ Down	−1.36	0.00		
	**WP_023464768.1**	**PA2533**	-	Probable sodium:alanine symporter	2	5	−1.08	0.26		−1.39	0.00		−1.57	0.00	↓ Down	−1.45	0.00		
	**WP_023464848.1**	**PA2777**	YfdC	Conserved hypothetical protein	3	5	1.15	0.03		1.04	0.66		−2.28	0.00	↓ Down	−4.83	0.00	↓ Lower	
	**WP_003452840.1**	**PA3236**	BetX	Betaine-specific binding protein	7	22	−1.07	0.02		−1.38	0.00		−3.35	0.00	↓ Down	1.40	0.00		
	**WP_003117607.1**	**PA3267**	-	Hypothetical protein	8	13	1.00	0.98		−1.29	0.00		−1.80	0.00	↓ Down	−1.20	0.01		
	**WP_003092370.1**	**PA3641**	-	Probable amino acid permease	5	25	−1.04	0.26		−1.40	0.00		−1.51	0.00	↓ Down	0.91	0.10		
	**WP_031636628.1**	**PA3709**	-	Probable major facilitator s↑ superfamily (MFS) transporter	6	19	−1.10	0.01		−1.12	0.00		−2.87	0.00	↓ Down	3.32	0.00	↑ Higher	
	WP_023465155.1	PA4023	Eat/EutP	Ethanolamine transporter, Eat	3	6	1.18	0.42		−1.83	0.09		−4.90	0.00	↓ Down	12.39	0.03	↑ Higher	
	WP_003105448.1	PA4503	DppB	Dipeptide ABC transporter permease DppB	3	5	−1.11	0.54		1.81	0.01	↑ Up	4.25	0.00	↑ Up				
	**WP_019726919.1**	**PA4710**	PhuR	Heme/Hemoglobin ↑ Uptake outer membrane receptor PhuR precursor	26	54	−1.11	0.00		−1.20	0.00		−3.12	0.00	↓ Down	1.34	0.00		
	**WP_003095599.1**	**PA4925**	-	Conserved hypothetical protein	2	4	−1.07	0.58		−1.18	0.17		−2.39	0.01	↓ Down	−7.73	0.00	↓ Lower	
	WP_003095948.1	PA5096	-	Probable binding protein component of ABC transporter	5	17	1.00	1.00		1.18	0.01		−2.31	0.00	↓ Down	4.11	0.00	↑ Higher	
	WP_003123634.1	PA5099	-	Probable transporter	4	10	1.05	0.39		1.07	0.14		−2.71	0.00	↓ Down	2.47	0.01	↑ Higher	
	WP_003105525.1	PA5167	DctP	DctP	16	74	1.04	0.35		1.54	0.00	↑ Up	−1.64	0.00	↓ Down	2.13	0.00	↑ Higher

These include proteins that were assigned to GO terms related to transmembrane transport and differentially regulated at the highest sub-MICs. The proteins were selected based on their assignment to at least one of the following GO terms: transmembrane transporter activity (GO:0022857), efflux transmembrane transporter activity (GO:0015562), transmembrane transport (GO:0055085), xenobiotic transport (GO:0042908), efflux pump complex (GO:1990281) or porin activity (GO:0015288). Orthologous proteins differentially regulated in both strains are in bold and shaded. Reg.: regulation.

### 3.6. Differentially regulated penicillin-binding proteins

PBPs, which are essential for the synthesis and maintenance of the peptidoglycan cell wall, are the targets of β-lactam antibiotics (Sauvage et al., 2008). Therefore, we looked at these proteins to determine which ones were differentially regulated in response to sub-MICs of meropenem in these two carbapenem-resistant strains (Table 6). In *P. aeruginosa*, at least eight PBPs have been described (Kong et al., 2010), of which two were differentially regulated, in both strains. Among those two was PBP3, one of the primary targets of meropenem (Kitzis et al., 1989; Davies et al., 2008), which previously has been shown to be indispensable for *P. aeruginosa* growth (Chen et al., 2017). PBP3 was upregulated in both strains, although at different levels. In strain CCUG 70744, PBP3 was moderately upregulated at the two highest sub-MICs (fold changes lower than 2). In strain CCUG 51971, PBP3 was also upregulated, but only at the highest sub-MIC and at a higher level (8.72-fold), which might be due to the shielding effect of the VIM-4 MBL (responsible for the high carbapenem resistance levels of this strain; Giske et al., 2003) at the two lowest sub-MICs. Additionally, in strain CCUG 70744, PBP3 had a higher basal abundance, compared to that of strain PAO1. Upregulation of PBP3 might contribute to the low susceptibilities of these strains, as overproduction of PBP3 in *P. aeruginosa* has been shown to reduce susceptibility to multiple β-lactam antibiotics (Liao and Hancock, 1997). In fact, mutations in PBP3 also have been associated with altered β-lactams susceptibilities, which highlights its relevance (Clark et al., 2019). A similar trend also was observed for PBP7, which in strain CCUG 70744 was moderately upregulated at the two highest sub-MICs (1.51- and 2.15-fold) while in strain CCUG 51971 it was highly upregulated but only at the highest sub-MIC (11.75-fold).

**Table 6:** Penicillin-binding proteins (PBPs) encoded by *Pseudomonas aeruginosa* CCUG 51971 and CCUG 70744 and their relative abundances.

							8/2 vs. 0 μg/ml			128/4 vs. 0 μg/ml			256/8 vs. 0 μg/ml			0 µg/ml VS CCUG 56489 (= PAO1) 0 µg/ml		
Strain	Product	Accession	PAO1 ortholog	Molecular weight (kDa)	# Peptides	# PSMs	Fold change	*p*-value	Reg.	Fold change	*p*-value	Reg.	Fold change	*p*-value	Reg.	Fold change	*p*-value	Basal abundance
CCUG 51971	Penicillin-binding protein 1a	WP_003095841.1	PA5045	91.2	23	47	1.08	0.30		−1.14	0.20		1.00	1.00					
	Penicillin-binding protein 1b	WP_003111352.1	PA4700	85.5	11	25	1.04	0.30		−1.17	0.01		1.33	0.00		1.28	0.01		
	Penicillin-binding protein 2	WP_003093191.1	PA4003	72.2	10	20	−1.32	0.35		1.28	0.26		1.35	0.80		1.39	0.27		
	**Penicillin-binding protein 3**	**WP_003094139.1**	**PA4418**	62.9	2	3	1.60	0.14		−1.40	0.35		8.72	0.00	↑ Up	−1.77	0.16		
	Penicillin-binding protein 3a	WP_003089297.1	PA2272	61.1	0	0													
	Penicillin-binding protein 4	WP_003091272.1	PA3047	51.9	12	36	−1.03	0.64		−1.09	0.20		−1.02	0.74		1.09	0.21		
	Penicillin-binding protein 5	WP_003093182.1	PA3999	42.5	13	83	−1.18	0.00		−1.16	0.00		1.22	0.01		1.24	0.01		
	**Penicillin-binding protein 7**	**WP_003085893.1**	**PA0869**	34	4	7	1.20	0.23		−1.17	0.31		11.75	0.00	↑ Up	−2.14	0.00	↓ Lower	
	**Possible penicillin-binding protein**	**WP_003084512.1**	**PA0378**	27.8	3	6	−2.08	0.00	↓ Down	1.16	0.16		1.89	0.00	↑ Up	1.70	0.00	↑ Higher	
	**Possible penicillin-binding protein**	**WP_023875768.1**	**PA0788**	116.4	16	30	−1.23	0.00		1.22	0.03		−3.58	0.00	↓ Down	1.05	0.26	
CCUG 70744	Penicillin-binding protein 1a	WP_003095841.1	PA5045	91.2	24	47	−1.02	0.23		−1.20	0.00		−1.27	0.00					
	Penicillin-binding protein 1b	WP_003100154.1	PA4700	85.5	12	24	1.07	0.11		−1.01	0.60		1.06	0.12		1.31	0.01		
	Penicillin-binding protein 2	WP_003093191.1	PA4003	72.2	9	16	−1.13	0.09		−1.30	0.09		−1.39	0.00		1.93	0.00	↑ Higher	
	**Penicillin-binding protein 3**	**WP_003094139.1**	**PA4418**	62.9	17	36	1.21	0.00		1.81	0.00	↑ Up	1.88	0.00	↑ Up	2.38	0.00	↑ Higher	
	Penicillin-binding protein 3a	WP_003110751.1	PA2272	61.1	0	0													
	Penicillin-binding protein 4	WP_003091272.1	PA3047	51.9	10	39	−1.02	0.58		−1.11	0.05		−1.06	0.15		1.00	0.93		
	Penicillin-binding protein 5	WP_003093182.1	PA3999	42.5	13	93	1.02	0.59		−1.10	0.04		−1.15	0.01		1.28	0.00		
	**Penicillin-binding protein 7**	**WP_003105974.1**	**PA0869**	34	3	4	1.07	0.52		1.51	0.05	↑ Up	2.15	0.02	↑ Up	−1.73	0.05	↓ Lower	
	**Possible penicillin-binding protein**	**WP_003100972.1**	**PA0378**	27.8	4	10	1.06	0.11		2.09	0.00	↑ Up	2.27	0.00	↑ Up	1.06	0.25		
	**Possible penicillin-binding protein**	**WP_023464312.1**	**PA0788**	116.4	18	26	1.00	0.84		−1.31	0.00		−2.47	0.00	↓ Down	1.23	0.02	

Orthologous proteins differentially regulated in both strains are in bold and shaded. Reg.: regulation.

Additionally, two possible PBPs, both containing a transglycosylase domain, were also differentially regulated. One of them (PA0378), annotated as “monofunctional biosynthetic peptidoglycan transglycosylase” by PGAP and as “probable transglycosylase” at the *Pseudomonas* Genome Database, was upregulated similarly to PBP3 and PBP7 in both strains, i.e., upregulated at the two highest sub-MICs in strain CCUG 70744 and only at the highest sub-MIC in strain CCUG 51971, although moderately. Strikingly, this putative PBP appeared downregulated at the lowest sub-MIC of strain CCUG 70744. The other one (PA0788), which is annotated as PBP by PGAP, was downregulated in both strains, at the highest sub-MIC. This might be relevant in the response to meropenem, as for instance, inactivation of PBP4 previously has been shown to cause β-lactam resistance by activating the CreBC two-component system and AmpC (Moya et al., 2009). Most of the other PBPs (PBP1a, PBP1b, PBP2, PBP4, and PBP5) were detected and quantitated in both strains, although none of them was differentially regulated. PBP3a was not detected in any of the strains.

### 3.7. Additional differentially regulated proteins related to peptidoglycan metabolism and cell wall organization

In addition to various PBPs, several other proteins related with peptidoglycan metabolism and cell wall organization, which can modulate tolerance and resistance to β-lactams (Cavallari et al., 2013; Monahan et al., 2014; Yadav et al., 2018), were differentially regulated (Table 7).

**Table 7:** Additional differentially regulated proteins related with peptidoglycan metabolism and cell wall organization.

							8/2 vs. 0 μg/ml			128/4 vs. 0 μg/ml			256/8 vs. 0 μg/ml			0 µg/ml VS CCUG 56489 (= PAO1) 0 µg/ml		
Strain	Accession	PAO1 ortholog	Gene name	Product	# Peptides	# PSMs	Fold change	*p*-value	Reg.	Fold change	*p*-value	Reg.	Fold change	*p*-value	Reg.	Fold change	*p*-value	Basal abundance
CCUG 51971	WP_003085647.1	PA0807	*ampDh3*	AmpDh3	7	16	−1.06	0.47		−1.22	0.03		−1.62	0.00	↓ Down	−2.15	0.00	↓ Lower
	WP_003093184.1	PA4000	*rlpA*	Endolytic peptidoglycan transglycosylase RplA	5	10	−1.10	0.94		1.30	0.45		16.68	0.00	↑ Up			
	WP_003132500.1	PA4001	*sltB1*	Soluble lytic transglycosylase B	6	14	1.17	0.27		−1.34	0.12		1.64	0.02	↑ Up	−1.16	0.51	
	WP_003093601.1	PA4201	*ddlA*	D-alanine-D-alanine ligase A	7	25	1.07	0.13		1.14	0.02		−1.69	0.00	↓ Down	−1.16	0.04	
	WP_003094120.1	PA4410	*ddlB*	D-alanine-D-alanine ligase	7	23	1.10	0.06		1.54	0.00	↑ Up	2.15	0.00	↑ Up	−1.34	0.00	
	WP_003094121.1	PA4411	*murC*	UDP-N-acetylmuramate–alanine ligase	9	27	1.04	0.40		1.31	0.00		1.83	0.00	↑ Up	−1.19	0.04	
	WP_003094133.1	PA4417	*murE*	UDP-N-acetylmuramoylalanyl-D-glutamate-2, 6-diaminopimelate ligase	16	47	1.09	0.05		1.01	0.70		1.50	0.00	↑ Up	−1.29	0.00	
	WP_003094332.1	PA4450	*murA*	UDP-N-acetylglucosamine 1-carboxyvinyltransferase	15	74	1.03	0.49		1.08	0.03		2.64	0.00	↑ Up	−1.20	0.00	
	WP_003095196.1	PA4749	*glmM*	Phosphoglucosamine mutase	15	73	1.12	0.05		1.59	0.00	↑ Up	1.82	0.00	↑ Up	1.01	0.85	
	**WP_003095661.1**	**PA4947**	***amiB***	N-acetylmuramoyl-L-alanine amidase	9	15	1.12	0.32		−1.60	0.01	↓ Down	3.14	0.00	↑ Up	2.26	0.01	↑ Higher
CCUG 70744	WP_023464890.1	PA2854	*erfK*	Putative L,D-transpeptidase	9	25	−1.13	0.02		1.57	0.00	↑ Up	2.19	0.00	↑ Up	−1.72	0.00	↓ Lower
	**WP_003123571.1**	**PA4947**	***amiB***	N-acetylmuramoyl-L-alanine amidase	12	16	−1.02	0.92		2.97	0.00	↑ Up	4.48	0.00	↑ Up	1.11	0.42	
	WP_003092734.1	-	*-*	D-alanyl-D-alanine dipeptidase	3	5	1.07	0.46		1.59	0.00	↑ Up	1.43	0.01				

The proteins were selected based on their assignment to at least one of the following GO terms: “peptidoglycan biosynthetic process” (GO:0009285), “peptidoglycan L,D-transpeptidase activity” (GO:0071972), “peptidoglycan glycosyltransferase activity” (GO:0008955), “peptidoglycan-based cell wall” (GO:0009274), “peptidoglycan catabolic process” (GO:0009253), “peptidoglycan turnover” (GO:0009254) or “cell wall organization” (GO:0071555). Orthologous proteins differentially regulated in both strains are in bold and shaded. Reg.: regulation.

In strain CCUG 51971, several of these were proteins involved in the peptidoglycan biosynthesis. Among these were the D-alanine-D-alanine ligases DdlA and DdlB, which demonstrated upregulation and downregulation, respectively; while DdlA appeared downregulated only at the highest sub-MIC, DdlB was upregulated at the two highest sub-MICs. Also, the amide ligases MurC and MurE and the enoylpyruvate transferase MurA, of the peptidoglycan biosynthesis pathway, were upregulated at the highest sub-MIC. The phosphoglucosamine mutase GlmM, crucial for the production of the cell wall precursor UDP-N-acetylglucosamine (Tavares et al., 2000), was also upregulated, in this case, at the two highest sub-MICs.

Several proteins involved in peptidoglycan catabolism also were differentially regulated. Those included two lytic transglycosylases (RlpA and SltB1; Blackburn and Clarke, 2002; Jorgenson et al., 2014) which are encoded by adjacent genes in the genome and were upregulated at the highest sub-MIC. The soluble lytic transglycosylase B (SltB1) was only slightly upregulated, although RlpA was highly upregulated (16.68-fold). The amidases AmpDh3 and AmiB were also differentially regulated. AmpDh3, which is one of the three AmpD homolog repressing the AmpC β-lactamase (Juan et al., 2006), was downregulated at the highest sub-MIC, which could then be at least partially responsible for the observed upregulation of the AmpC PDC-35. Its orthologue in strain CCUG 70744 was not detected. The other AmpD homolog (AmpD and AmpDh2) were detected but not differentially regulated, except for AmpD in strain CCUG 51971, which was not detected.

AmiB, which is crucial for cell division and its deletion has been shown to affect the outer membrane integrity, which increases its permeability and causes hyper-susceptibility to multiple antibiotics, including meropenem (Yakhnina et al., 2015), appeared slightly downregulated at 128 μg/ml but with clear upregulation at 256 μg/μl. Interestingly, AmiB was also upregulated at relatively high levels in strain CCUG 70744, in this case, at the two highest sub-MICs.

In strain CCUG 70744, only two other proteins related to peptidoglycan metabolism and cell wall organization, apart from AmiB, were differentially regulated. One was ErfK, a putative L,D-transpeptidase, possibly involved in peptidoglycan cross-linking, which was upregulated at the two highest sub-MICs. Because of its possible L,D-transpeptidase activity, the upregulation of ErfK could potentially compensate the harmful effects of meropenem, which blocks the D,D-transpeptidase activity of PBPs, essential for peptidoglycan cross-linking (Aliashkevich and Cava, 2022). The other protein was a D-alanyl-D-alanine dipeptidase, possibly involved in peptidoglycan catabolism, which was slightly upregulated at 4 μg/μl but did not reach the significance thresholds at 8 μg/ml.

### 3.8. Differentially regulated regulatory proteins

Using the webserver P2RP, a total of 599 and 597 regulatory proteins (including two-component systems, transcription factors and other DNA binding proteins) were predicted in the carbapenem-resistant strains CCUG 51971 and CCUG 70744, respectively. These represent 8.6% and 9.3% of their respective theoretical proteomes, which is in accordance with what was previously reported for strain PAO1 (Stover et al., 2000). Of all predicted regulatory proteins, 359 and 388 were detected and quantitated and, of those, 189 and 79 had significant changes in their relative abundance in at least one condition, respectively (Table 8). All regulatory proteins that were differentially regulated in at least one condition are presented in Supplementary Tables 4, 5.

**Table 8:** Number of regulatory proteins predicted by P2RP for each strain and number of those that were differentially regulated in at least one condition.

		CCUG 51971		CCUG 70744	
Category	Class	Total predicted	Differentially regulated^a^	Total predicted	Differentially regulated^a^
Two-component systems	Histidine kinases	62	28	64	10
	Response regulators	72	36	75	13
	Phosphotransfer proteins	5	0	5	0
Transcription factors	Transcriptional regulators	205	51	199	19
	One-component systems	188	55	187	29
	Response regulators	48	21	51	9
	Sigma factors	26	3	25	1
Other DNA-binding proteins	Other DNA-binding proteins	41	16	42	7

a In at least one condition.

In both carbapenem-resistant strains, a significant proportion of regulatory proteins were differentially regulated. For instance, in strain CCUG 51971, a 45%–50% of all the predicted two-component systems, which play a critical role in controlling how the cells respond to environmental stimuli, were differentially regulated (the majority downregulated), most of them at 256 μg/ml. This proportion was much lower in strain CCUG 70744, but still, the large number of regulatory proteins that presented changes in their relative abundances, especially at high sub-MICs, already suggest that the responses of the cells to these sub-MICs are vast, as it has been demonstrated by the large number of differentially regulated proteins determined in both strains. This warrants future studies aiming to confirm and elucidate the role of these two-component systems in the responses of *P. aeruginosa* to carbapenems.

Several sigma factors were differentially regulated in response to meropenem. In strain CCUG 51971, AlgU, which is involved in alginate biosynthesis and thus conversion to mucoid phenotype (Martin et al., 1993), and in resistance to oxidative and heat-shock stress (Martin et al., 1994), was upregulated at 256 μg/ml (1.63-fold). The sigma factor SbrI, which controls swarming motility and biofilm formation (McGuffie et al., 2016), appeared moderately downregulated at 128 ng/μl (−1.63-fold), but below although close to the threshold of significance at 256 μg/ml (−1.48-fold). A third differentially regulated sigma factor was *FliA*, which controls the synthesis of flagellin (the main component of flagella; Starnbach and Lory, 1992) and was downregulated at 256 μg/ml (−2.52-fold). In strain CCUG 70744, FiuI, which is involved in ferrichrome-mediated iron up-take (Llamas et al., 2006), was upregulated at 4 μg/ml (1.55-fold) but not at 8 μg/ml.

Among all predicted regulatory proteins, we specifically looked at AmpR (PA4109), a transcription factor that, in addition to proteases, quorum sensing and virulence factors, regulates the naturally-ocurring AmpC β-lactamase (Kong et al., 2005), which was upregulated in both strains. In strain CCUG 70744, AmpR was detected and quantitated, but not differentially regulated in any condition, even though AmpC was highly upregulated at 128 and 256 μg/ml. Comparison with strain PAO1 showed no differences in the basal abundances of AmpR in strain CCUG 70744. In strain CCUG 51971, AmpR was detected but not quantitated, which might be due to low protein abundance.

Also worth highlighting is the fact that CreD, which is the effector protein of the CreBC two-component system and plays a major role in bacterial fitness and biofilm formation, especially in the presence of β-lactam antibiotics (Zamorano et al., 2014), was upregulated in all three conditions in strain CCUG 70744 (WP_003084736.1; Supplementary Table 3). In strain CCUG 51971, CreD was upregulated at high levels (6.03-fold) but only at the highest sub-MIC (WP_003084736.1; Supplementary Table 2). The activation of CreBC, reflected by CreD, has been shown to be a major β-lactam resistance driver (Moya et al., 2009; Zamorano et al., 2014). Activation of CreBC has been previously shown to be triggered by the inactivation or inhibition of PBP4 (Moya et al., 2009; Zamorano et al., 2014). PBP4 is one of the PBP for which meropenem has its highest affinity (Davies et al., 2008). Therefore, the activation of CreBC in both strains might be caused by PBP4 inhibition by meropenem. Indeed, strong expression of *creD* in response to imipenem and meropenem has been observed in other studies [(Bagge et al., 2004) and GEO, Gene Expression Omnibus, dataset GSE167137]. The relative abundance of PBP4 did not vary in any of the strains in response to meropenem (Table 6). However, inhibition does not necessarily result in a decrease in the protein abundance. It is also worth noting that, although the activation of a two-component system such as CreBC does not necessarily imply changes in its relative abundance, CreB (WP_003084734.1) was slightly downregulated at the highest concentration in strain CCUG 51971 (−1.55-fold) and CreC (WP_003084735.1) was below but close to the threshold of significance (−1.47-fold), which may also have implications in the response of the bacterium to high sub-MICs of meropenem.

### 3.9. Differentially regulated proteins of unknown function

Despite *P. aeruginosa* being a well characterized bacterium, the carbapenem-resistant strains CCUG 51971 and CCUG 70744 encode 1,139 and 1,251 hypothetical proteins (according to the PGAP v4.9 and v4.5 annotations available in RefSeq), respectively (i.e., 17.6% and 19.6% of the theoretical proteomes). Hundreds of these hypothetical proteins (307 and 345, respectively) were detected by the nano-LC–MS/MS analyses, which implies that they are not hypothetical, and that, from now on, should be considered “proteins of unknown function.” Additionally, both strains also encode numerous proteins with domains of unknown function (DUF; 341 and 362), of which 143 and 142 were detected, respectively.

Interestingly, many of these proteins of unknown function were also upregulated or downregulated in at least one condition (244 in strain CCUG 51971 and 152 in strain CCUG 70744), suggesting that they are relevant in the response of these strains to meropenem and that they deserve further investigation to elucidate their functions and possible relation with antibiotic resistance. Moreover, 41 and 46 of them were upregulated or downregulated in at least two sub-MICs, respectively. All proteins annotated as “hypothetical proteins” or containing DUFs that were differentially regulated in at least one condition are presented together with their functional annotation (assignment to COG categories, GO terms, Pfam families, Enzyme Comission numbers and InterPro IDs) in Supplementary Tables 6, 7.

### 3.10. Upregulation of a type VI secretion system

**Figure 3:**
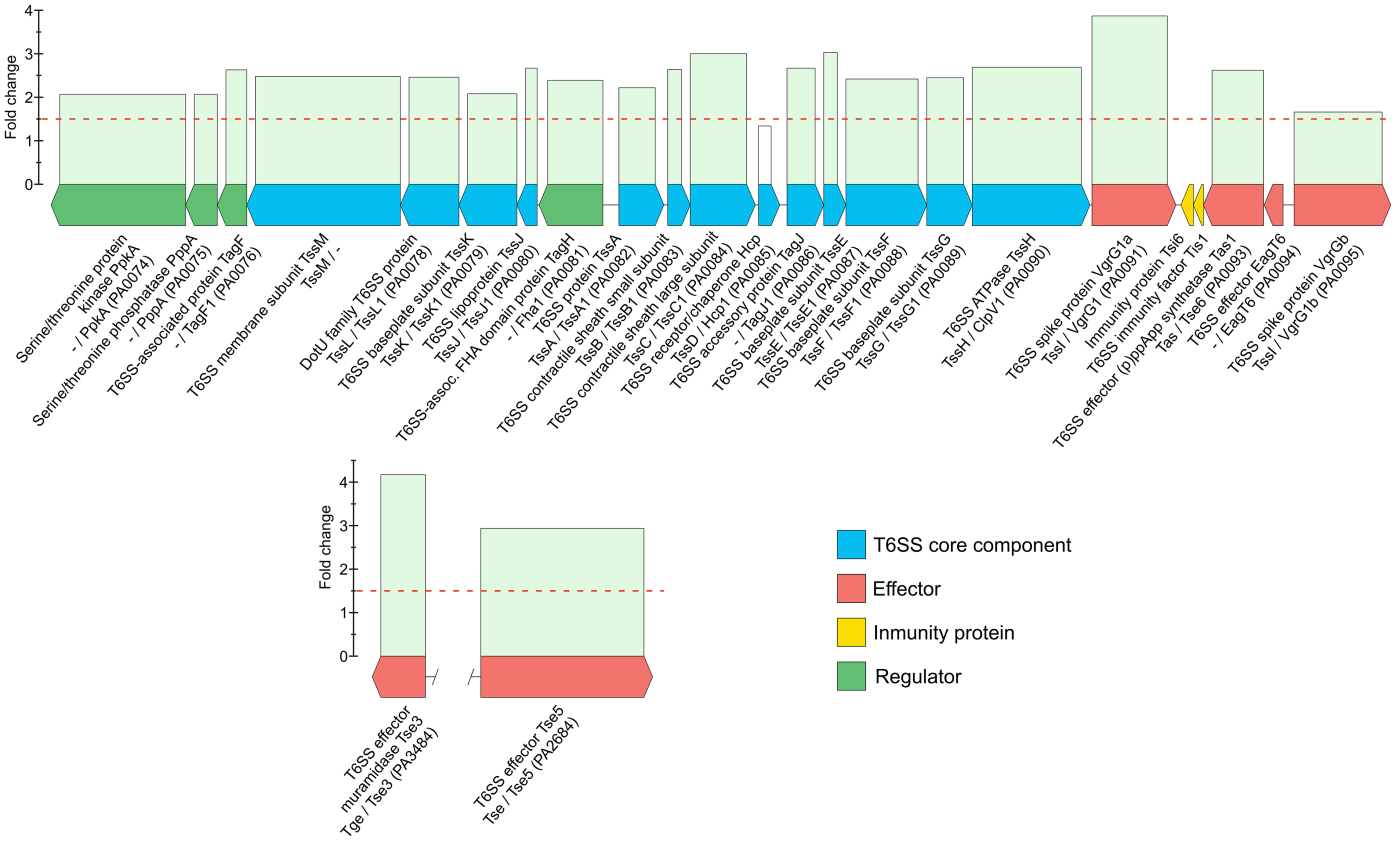
Genetic map of the H1-T6SS of *Pseudomonas aeruginosa* CCUG 51971 and relative abundances of its protein products at 256 μg/ml (½ MIC) compared to 0 μg/ml. Annotations from RefSeq, SecReT6 and *Pseudomonas* Genome Database are indicated, if available.

The genome of *P. aeruginosa* harbors three T6SSs, named H1-T6SS to H3-T6SS (Hood et al., 2010), which are bacterial multiprotein devices capable of targeting effectors into prokaryotic and eukaryotic cells (Ho et al., 2014). Remarkably, all of the 14 described core components and several other associated proteins of the H1-T6SS, were upregulated in the carbapenem-resistant strain CCUG 51971 at the highest sub-MIC (256 μg/ml; Figure 3; Supplementary Table 8). These also included five effector proteins predicted for this system by the software SecReT6: the spike-carriers VgrG1 and VgrG1b and the toxins Tse3, Tse5, and Tse6. Interestingly, four of these effectors were also upregulated at 128 μg/ml and two also at 8 ng/μl. Although these toxins target bacterial cells (Hood et al., 2010; Hachani et al., 2014; Whitney et al., 2014), the H1-T6SS also has been shown to be active and important for the fitness of *P. aeruginosa* during chronic infections (Potvin et al., 2003; Mougous et al., 2006), which implies that the upregulation of this system in response to certain sub-MICs of meropenem may have direct implications in the virulence of the strain, either by outcompeting other cells or by directly affecting also the host, as has been shown for other T6SSs of *P. aeruginosa* (Sana et al., 2016).

Additionally, the H1-T6SS has been associated with decreased susceptibility to various antibiotics in biofilms (Zhang et al., 2011) and most of the proteins of the H1-T6SS of strain CCUG 51971 also had a higher basal relative abundance than their homolog in PAO1, suggesting that this system could potentially play a role in the resistance of this strain to multiple antibiotics. Indeed, 22 out of 23 predicted components and effectors of H1-T6SS of strain CCUG 51971 that were compared with the publicly available transcriptomic dataset in which biofilms of *P. aeruginosa* PAO1 were treated with 5 μg/ml of meropenem (GEO accession number: GSE167137), appeared also upregulated in that study. Furthermore, the TagR1 iModulone (i.e., independently modulated set of genes) that encompasses the H1-T6SS (Rajput et al., 2022b) was recently observed to be upregulated in response to various β-lactam antibiotics, including meropenem (Rajput et al., 2022a). Therefore, future studies aiming to validate this response as well as to determine the potential impact on virulence and antibiotic resistance, and if there is any advantage for the bacterium in upregulating this T6SS in response to high sub-lethal concentrations of meropenem, are warranted and would be of great value.

In strain CCUG 70744, 11 of the core components of the H1-T6SS were detected and eight were quantitated, but only one was significantly upregulated (TssB, PA0083) together with an effector protein (EagT6, PA0094). This suggests that in this strain the system is expressed but that it might only be subtly or not upregulated in response to sub-MICs of meropenem.

### 3.11. Clusters of orthologous groups enrichment analysis

Proteins that were differentially regulated in the cultures with the two highest sub-MICs of meropenem (i.e., ½ and ¼ of the MIC), were classified into COG categories, to determine if any category was significantly upregulated or downregulated and/or significantly enriched.

**Figure 4:**
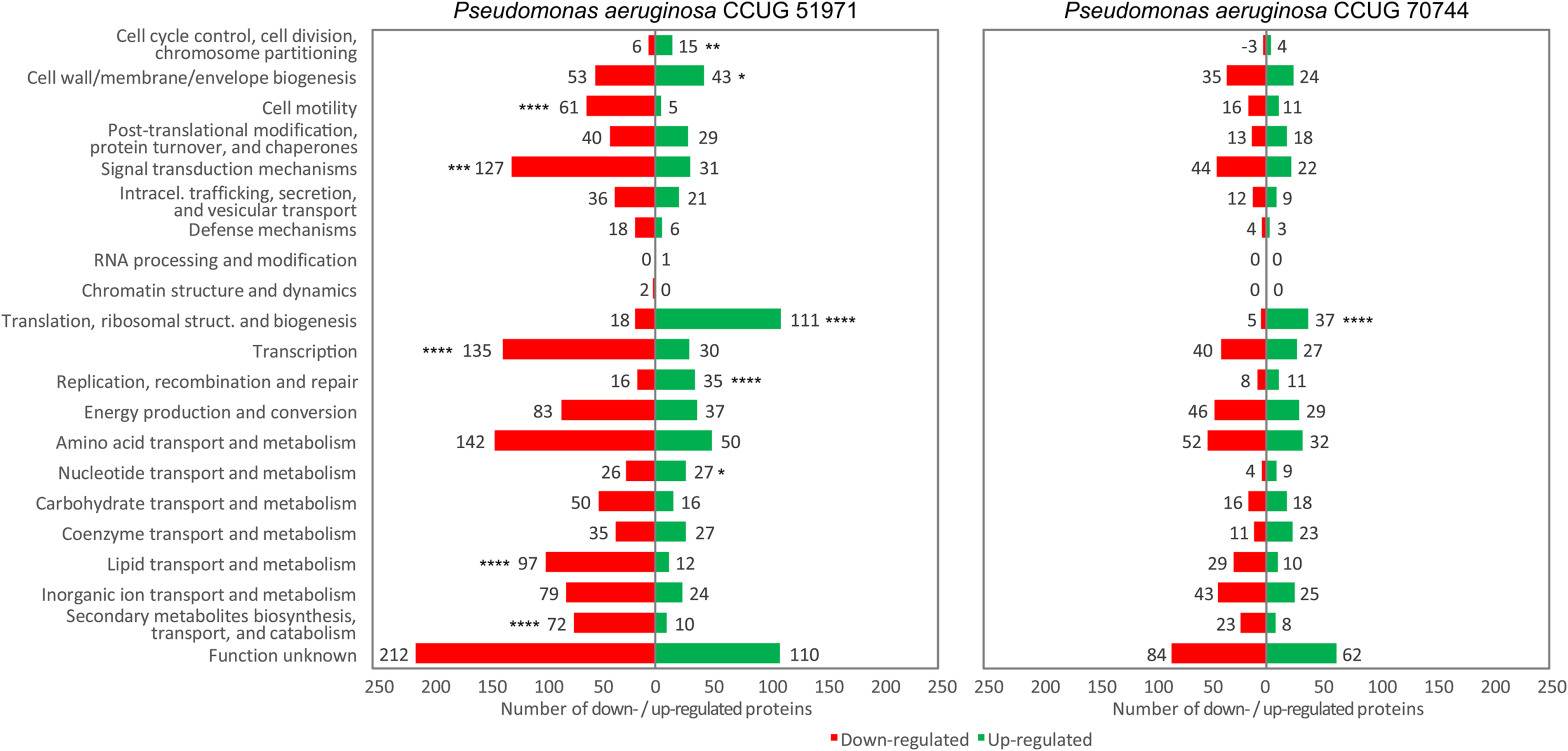
Functional classification (into COG categories) of the proteins that were upregulated or downregulated in the samples cultivated with the highest sub-MIC of *Pseudomonas aeruginosa* CCUG 51971 (256 μg/ml) and CCUG 70744 (8 μg/ml). Significantly upregulated or downregulated COG categories are marked as follows: * if *p*-value < 0.05; ** if *p*-value < 0.005; *** if *p*-value < 0.001; **** if *p*-value < 0.0001.

The analysis revealed that, in both carbapenem-resistant strains, most of the differentially regulated proteins within the category “Translation, ribosomal structure and biogenesis” were upregulated (Figure 4). Upregulation of genes associated with translation and ribosomal proteins has been previously reported in response to antimicrobial agents, suggesting an increase in protein production as a protective response (Nde et al., 2008; Sung et al., 2021; Rajput et al., 2022a). In strain CCUG 51971, most proteins within the category “Transcription” were downregulated, although several RNA polymerase subunits were upregulated (RpoB, RpoC, and RpoZ).

In strain CCUG 70744, no other categories were significantly upregulated or downregulated. However, in strain CCUG 51971, most of the differentially regulated proteins in the following COG categories were upregulated: “Nucleotide transport and metabolism,” “Replication, recombination and repair” (which was also upregulated at 128 μg/ml), “Cell cycle control, cell division, chromosome partitioning” and “Cell wall/membrane/envelope biogenesis.” Meanwhile, most of the differentially regulated proteins within the following categories were downregulated: “Signal transduction mechanisms,” “Lipid transport and metabolism,” “Secondary metabolites biosynthesis, transport, and catabolism” and “Cell motility.” Of these, the last three were also significantly downregulated at 128 μg/ml.

Additionally, the analysis in strain CCUG 51971 revealed that, when compared with all quantitated proteins, the COG categories “Signal transduction mechanisms,” “Translation, ribosomal structure and biogenesis,” “Secondary metabolites biosynthesis, transport, and catabolism” and “Cell motility” were significantly enriched (i.e., had a higher proportion of differentially regulated proteins) at 256 μg/ml. Additionally, “Cell motility” was also enriched at 128 μg/ml. In strain CCUG 70744, none of those categories was enriched, but “Energy production and conversion” and “Inorganic ion transport and metabolism” were significantly enriched at 8 μg/ml.

### 3.12. Gene ontology terms and pathway enrichment analyses

**Figure 5:**
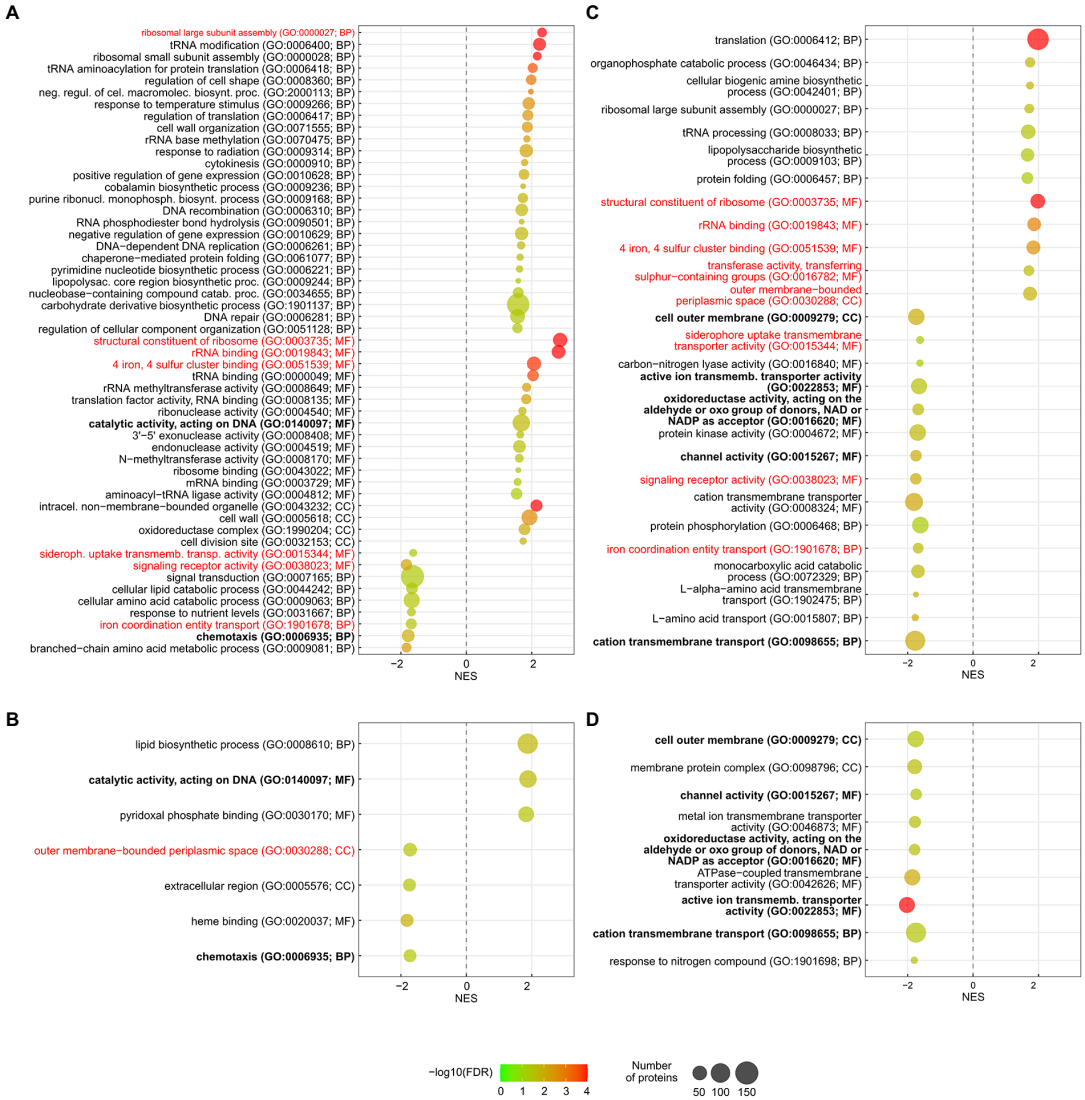
GO terms that were significantly enriched when the strains were cultivated with the two highest sub-MICs of meropenem. *Pseudomonas aeruginosa* CCUG 51971 was cultivated with 256 μg/ml **(A)** and 128 μg/ml **(B)**. *P. aeruginosa* CCUG 70744 was cultivated with 8 μg/ml **(C)** and 4 μg/ml **(D)**. In red: GO terms enriched in both strains. In bold: GO terms enriched in both conditions of one strain. BP, biological process; MF, molecular function; CC, cellular component; FDR, false discovery rate; NES, normalized enrichment score.

**Figure 6:**
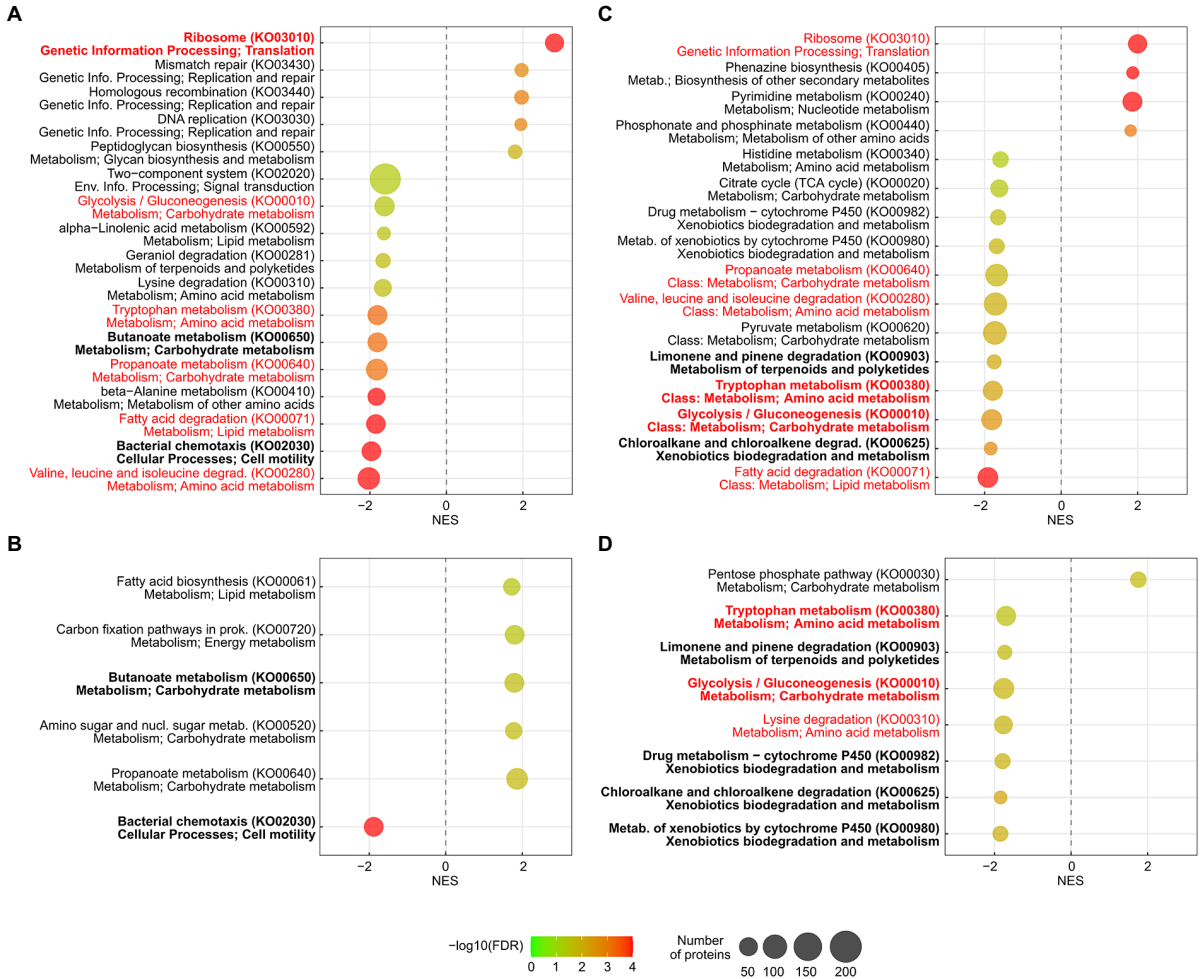
KEGG pathways that were significantly enriched when the strains were cultivated with the two highest sub-MICs of meropenem. *Pseudomonas aeruginosa* CCUG 51971 was cultivated with 256 μg/ml **(A)** and 128 μg/ml **(B)**. *P. aeruginosa* CCUG 70744 was cultivated with 8 μg/ml **(C)** and 4 μg/ml **(D)**. The row below each pathway indicates its class. In red: pathways enriched in both strains. In bold, pathways enriched in both conditions of one strain. FDR, false discovery rate; NES, normalized enrichment score.

To determine if more specific subsets of proteins were significantly enriched, proteins were assigned to gene ontology (GO) terms, KEGG pathways were annotated and GSEAs were performed. In the cases of both carbapenem-resistant strains, numerous GO terms were significantly upregulated or downregulated at the highest sub-MIC and multiple upregulated GO terms were related with translation and with ribosomal assembly (Figure 5), including those with the highest normalized enrichment scores (NES), supporting the results of the COG enrichment analysis. Indeed, Ribosome (KO03010) was the upregulated pathway with the highest NES in both strains. Additionally, several cell wall metabolism and outer membrane-related GO terms and pathways were significantly enriched in both strains, which could be expected considering that the cell wall is the main target of β-lactam antibiotics. Moreover, the lipopolysaccharide core region biosynthetic process (GO:0009244) was upregulated in both strains at the highest sub-MIC, which may have important implications in the virulence of the cells during infection (Pier, 2007). Moreover, several transport-related GO terms were downregulated in both strains, but more abundantly in strain CCUG 70744, which reflects a response to decrease the permeability of the cells, a well-known protection mechanism against carbapenems (Blair et al., 2015). In both strains, signaling receptor activity appeared downregulated, although most of these proteins were associated with chemotaxis and reception of siderophores. Indeed, siderophore uptake transmembrane transporter activity (GO:0015344) and iron coordination entity transport (GO:1901678) were also downregulated with most core enrichment sequences being siderophore receptors, which could potentially be a protective response to prevent the intake of sideromycins. This may have important metabolic implications, as iron is critical for bacterial growth and survival but, certain conditions such as oxidative stress can lead to intracellular iron release and this to generation of cytotoxic reactive oxygen species (Frawley and Fang, 2014). Additionally, chemotaxis appeared downregulated in strain CCUG 51971, at the two highest sub-MICs. Indeed, 29 of the 44 proteins assigned to Chemotaxis (GO:0006935) were downregulated at the highest sub-MIC, a response which probably aims to reduce mobility and promote biofilm formation. This response is in line with the downregulation of the sigma factors SbrI and *FliA*, which are associated with motility (Starnbach and Lory, 1992; McGuffie et al., 2016). Moreover, in strain CCUG 51971, various stress-related GO terms (response to temperature stimulus, radiation, DNA recombination, DNA repair) were upregulated at the highest sub-MIC, suggesting that the highest sub-MIC triggered a strong stress response in this strain. Indeed, this is reflected by the upregulation of several prophage-associated proteins, which might indicate that prophage induction was triggered by 256 μg/ml. It is also in line with the upregulation of the sigma factor AlgU, which is associated with alginate production and resistance to oxidative and heat-shock stress (Martin et al., 1993, 1994). A previous study showed that exposure of *P. aeruginosa* biofilms to imipenem induced alginate biosynthetic genes, which may be an adverse consequence of imipenem treatments (Bagge et al., 2004). However, proteins of those genes were not detected in this study (Figure 6).

Interestingly, numerous metabolic pathways were significantly enriched in both strains, with most of these being downregulated at the highest sub-MIC, affecting in both cases metabolism of carbohydrates, amino acids and lipids. Metabolic adaptation was recently shown to be able to cause antibiotic resistance (Lopatkin et al., 2021) and in fact, multiple metabolic genes of *P. aeruginosa* have been associated with alterations in antibiotic susceptibility (Fajardo et al., 2008). This warrants future studies aiming to elucidate the impact of metabolic responses of *P. aeruginosa* in antibiotic resistance.

### 3.13. Strengths and limitations of the study

MS-based proteomic approaches, in combination with whole-genome sequencing, offer the advantage of directly detecting produced proteins or peptides. In this study, we have shown that quantitative shotgun proteomics, using TMT labeling and nano-LC–MS/MS analysis, is an effective approach for detecting the global and specific responses of *P. aeruginosa* strains to sub-MICs of meropenem, by determining differentially regulated proteins, COG categories, GO terms and pathways. Moreover, it can be used to determine differences in the basal relative abundances of highly homologous proteins in different strains.

An inherent limitation of shotgun MS-based proteomic approaches is that, even though the techniques have advanced significantly over the last years, they still cover only a fraction of the total proteome (Meyer et al., 2011). This is due to the lack of or low expression of certain genes at certain growth conditions, but also due to limitations of the enzymatic protein digestion (trypsin has to be capable of digesting a protein in order to generate peptides; Giansanti et al., 2016), separation capacity of the liquid chromatography and inherent limitations of the mass spectrometry analysis techniques employed, such as the use of quantitative multiplexed data (Pappireddi et al., 2019) or use of data dependent acquisition (Li et al., 2020). For instance, highly hydrophobic proteins (e.g., some membrane proteins) typically contain lower numbers of positively charged arginine or lysine residues, which are the targets of trypsin. Therefore, some of these proteins typically generate less peptides that are suitable for LC–MS/MS detection (Alfonso-Garrido et al., 2015). This drawback is reflected in this study, where we observed that, 55.5% and 54.9% of the theoretical proteomes of the strains were detected, but only 42.0% and 38.9% of the proteins assigned to the GO term “integral component of membrane” (GO:0016021), respectively, were detected.

Altogether these limitations affect the coverage of an actual proteome and imply that proteins being produced and playing a significant role in the response of the strains to meropenem might have been missed due to lack of detection or quantitation. But overall, in this study, 55.5% and 54.9% of the accessioned proteins were detected and 50.4% and 51.0% quantitated. Transcriptomic approaches can provide expression levels for most genes being expressed and, hence, may sometimes be perceived as a more comprehensive and less cumbersome alternative. However, low correlation between transcriptomic and proteomic data has been observed and can occur due to multiple reasons (Haider and Pal, 2013), whereas proteins are the ultimate effectors of the organism. Thus, quantitative proteomics represents a more direct approach for observing the responses of cells, although a good approach could also be to apply multiple approaches, such as shotgun genomic, transcriptomic and proteomic analyses, as proposed by Hua et al. (2017). In any case, although many proteins might not be generated at all or produced at very low levels, we were able to detect and quantitate more than half of the theoretical proteome, which provides a comprehensive image of the response of the bacteria to sub-MICs of meropenem.

It is also important to consider that the theoretical proteomes of the strains included in this study were determined, using the NCBI annotation pipeline PGAP (Tatusova et al., 2016). This implies that, even though *P. aeruginosa* is a significant bacterial pathogen (Ikuta et al., 2022) and relatively well-characterized bacterium (Stover et al., 2000; Winsor et al., 2016), errors may exist in the annotations. Thus, the actual existence, the accurate sequence, and the length of multiple annotated but yet uncharacterized proteins is often uncertain. Experimental proteomic data, such as those generated in this study, can be of great value for addressing this problem in further studies, by performing proteogenomics (i.e., genome annotation guided by proteomic data; Armengaud et al., 2014).

Another limitation of isobaric labeling-based approaches is that normally they can only detect changes in relative abundances but cannot do absolute quantification. Thus, proteins encoded by genes that have been constitutively under or overexpressed but that do not vary between conditions, will not be noted, even though those high or low levels of abundance might be involved in resistance. However, this issue can be addressed by including strains with highly similar orthologous sequences, as in this case PAO1. For instance, no changes in the relative abundance of PIB-1 nor MexXY were detected in strain CCUG 70744, but the comparison with strain PAO1 revealed that they had significantly higher basal abundances, which has previously been associated with β-lactam resistance (Masuda et al., 2000; Fajardo et al., 2014).

An important consideration when performing proteomic studies is the time point when the samples are collected. In this study, we used 20 h cultivation time, because antibiotic concentrations affect the growth dynamics of bacterial cells; the cultures were incubated for 20 h, similar to what has been reported in other studies (Wang et al., 2019; Kong et al., 2021), when satisfactory growth had been obtained for all the cultures, i.e., they were at a similar and late stage and had been exposed to meropenem for the same time. However, studies aiming to detect early responses may be designed with shorter incubation times. Studies comparing different time points would also be of interest to elucidate the dynamics of responses to meropenem.

Additional variables of the proteomic study design are the fractions of the cells that are analyzed. While, in this study, we attempted to analyze the whole cell proteome, alternative studies focusing on analyzing specific fractions, such as the surface proteome or the exoproteome, to determine the responses of surface proteins or exoproteins (i.e., exported proteins and proteins resulting from cell lysis or leakage), which are in direct contact with the environment surrounding the cells, will be important for understanding site-specific responses (Armengaud et al., 2012; Wolden et al., 2020). In any case, our study illustrates the potential of quantitative proteomic approaches to reveal global and specific changes in the proteomes of carbapenem-resistant *P. aeruginosa* strains with different mechanisms of resistance, in response to antibiotic stress. We have detected numerous proteins that, at least in the context of these two carbapenem-resistant *P. aeruginosa* strains, are differentially regulated when cultivated with sub-MICs of meropenem. This may serve as an important guide for future studies aiming to confirm the roles of these proteins in the response of *P. aeruginosa* to meropenem. It will also serve as a basis for future investigations searching alternative resistance targets and low-level resistance mechanisms. However, an obvious limitation of this study is that only two carbapenem-resistant strains of only two high-risk clones have been used, while *P. aeruginosa* is a diverse species with an exceptionally large and open pan-genome (Freschi et al., 2019). This means that the analysis of additional strains with different genetic backgrounds will most probably reveal new and different responses.

Indeed, multiple different responses to sub-lethal concentrations of meropenem have been observed in this study by including just two carbapenem-resistant strains. For instance, while strain CCUG 70744 responded to all three sub-lethal concentrations of meropenem by upregulating PDC-8, strain CCUG 51971 upregulated PDC-35 only at the highest concentration. This could be due to a protective effect of the VIM-4 MBL, which is upregulated only at 256 μg/ml, but to a high level. Notable differences were also observed among porins, such as OprD, which was truncated in strain CCUG 51971, and downregulated in strain CCUG 70744 at 4 and 8 μg/ml. Both strains downregulated multiple other porins at the highest sub-lethal concentrations. However, at one fourth of the MIC, only one porin appeared to be downregulated in strain CCUG 51971, while 12 porins were downregulated in the case of strain CCUG 70744. Further differences were observed regarding efflux pumps. For instance, while both strains presented higher basal abundances of MexXY compared to strain PAO1, upregulation of this efflux pump in response to meropenem was observed only in strain CCUG 51971. It is also worth noting that, among PBPs, PBP3 and PBP7 were upregulated in both strains. However, while both PBPs in strain CCUG 70744 were upregulated at the two highest sub-MICs, and at moderate levels, in strain CCUG 51971, both PBPs were upregulated only at the highest sub-MIC, but at high levels, which could, again, potentially be due to the protective effect of VIM-4 at lower concentrations. It is also worth noting that while multiple pathways and groups of proteins were upregulated or downregulated in both strains (e.g., glycolysis/gluconeogenesis, KO00010, which was downregulated in both strains), others were observed to be upregulated or downregulated only in one strain (e.g., chemotaxis, KO02030, downregulated only in strain CCUG 51971, at the two highest sub-MIC levels).

Thus, it will be important to look at how frequent and conserved these differentially regulated proteins are among other strains of *P*. *aeruginosa*, to determine if they are relevant to only a few strains or to a larger proportion of the species. This could be done by screening the >7,000 publicly available genome sequences of *P. aeruginosa*, although further experiments confirming the involvement of these proteins in resistance to meropenem in numerous strains should be performed. Thus, future studies including more strains with different and diverse genomic backgrounds and acquired resistance mechanisms, as well as additional antimicrobial compounds and conditions (e.g., *in vivo* models), will be essential to determine conserved putative alternative resistance mechanisms in *P. aeruginosa*. and to elucidate further responses.

## 4. Conclusion

The exposure of the carbapenem-resistant *P. aeruginosa* strains CCUG 51971 (ST235) and CCUG 70744 (ST395) to sub-MICs of meropenem caused significant strain-and concentration-specific changes in their proteomes. The marked responses involve complex interactions between multiple canonical and non-canonical mechanisms, each of which might be accountable at different levels for the low susceptibility of these strains to carbapenem antibiotics. Additionally, multiple proteins of unknown function were differentially regulated in both strains, in response to exposure to sub-MIC levels of meropenem, which warrants future studies to explore their function and determine if and how they are involved in the responses of these strains to meropenem. Our study demonstrates that quantitative shotgun proteomics is effective for identifying different and complex mechanisms that collectively may play a role in reduced susceptibility of *P. aeruginosa* to meropenem. Our study provides a framework for elucidating responses in expression and determining alternative mechanisms of resistance on exposure to antibiotics, that may lead to identifying new drug targets in pathogens.

## Data availability statement

The strains used in this study are available at the Culture Collection University of Gothenburg (CCUG, Gothenburg, Sweden; www.ccug.se). The complete genome sequence of *P. aeruginosa* CCUG 51971 (= PA 66) has been deposited in DDBJ/ENA/GenBank under the accession no. CP043328. The complete genome sequence of *P. aeruginosa* CCUG 70744 was already publicly available in DDBJ/ENA/GenBank under the accession no. CP023255. The Illumina and Oxford Nanopore sequence reads of *P. aeruginosa* CCUG 51971 have been deposited in the Sequence Read Archive (SRA) (Leinonen et al., 2011), under the accession numbers SRX6772487, SRX6772488 and SRX6772489. The mass spectrometry proteomics data has been deposited in the ProteomeXchange Consortium (Deutsch et al., 2019) via the PRIDE partner repository (Perez-Riverol et al., 2019) with the dataset identifier PXD034987.

## Author contributions

FS-S, IA, EM, and RK: conceptualization. FS-S, DJ-L, and RK: methodology. FS-S, DJ-L, NM, IA, and RK: validation. FS-S: original draft preparation. FS-S, DJ-L, NM, IA, EM, and RK: review and editing. EM and RK: supervision and project administration. DJ-L, IA, EM, and RK: funding acquisition. All authors contributed to the article and approved the submitted version.

## Funding

This study was supported by the Centre for Antibiotic Resistance Research (CARe) at the University of Gothenburg (project no: 5314-205314021), Laboratoriemedicin FoU (project no. 51060-6268), the Swedish Västra Götaland regional funding (projects no. ALFGBG-437221 and ALFGBG-720761), and by the Culture Collection University of Gothenburg (CCUG; www.ccug.se) Project: Genomics and Proteomics Research on Bacterial Diversity. The CCUG was supported by the Department of Clinical Microbiology, Sahlgrenska University Hospital, Gothenburg, Region Västra Götaland, Sweden. The computations were partially performed on resources provided by the Swedish National Infrastructure for Computing (SNIC) through the Uppsala Multidisciplinary Center for Advanced Computational Science (UPPMAX) under project SNIC 2019/8-176.

## Conflict of interest

RK was affiliated to the company Nanoxis Consulting AB. The company did not have influence on the conception, elaboration, and decision to submit the present research article.

The remaining authors declare that the research was conducted in the absence of any commercial or financial relationships that could be construed as a potential conflict of interest.

## Publisher’s note

All claims expressed in this article are solely those of the authors and do not necessarily represent those of their affiliated organizations, or those of the publisher, the editors and the reviewers. Any product that may be evaluated in this article, or claim that may be made by its manufacturer, is not guaranteed or endorsed by the publisher.

## Footnotes

^1^ https://www.eucast.org/clinical_breakpoints/

## Supplementary material

SUPPLEMENTARY FIGURE 1 Heatmap representations and dendrograms of the quantitative nano-LC-MS/MS-based proteomic data of *Pseudomonas aeruginosa* CCUG 51971 (a) and *P. aeruginosa* CCUG 70744 (b). For each strain, the four replicates of each of the four conditions are included.

SUPPLEMENTARY FIGURE 2 Principal component analysis (PCA) plots, based on the results of the quantitative nano-LC-MS/MS-based proteomic analyses of the quadruplicate samples of: **a** *Pseudomonas aeruginosa* CCUG 51971; and **b** *P. aeruginosa* CCUG 70744, exposed to different sub-MICs of meropenem (strain CCUG 51971: 8, 128 and 256 μg/ml; strain CCUG 70744: 2, 4 and 8 μg/ml) and to no antibiotic. Each plot shows four groups of samples (one per meropenem concentration), distributed according to their similarity by principal components (PC) 1 and 2. Each dot represents one replicate.

SUPPLEMENTARY FIGURE 3 Volcano plots of the six quantitative proteomic comparisons. **a** *Pseudomonas aeruginosa* CCUG 51971 at 256 μg/mL compared to 0 μg/mL. **b** *P. aeruginosa* CCUG 51971 at 128 μg/mL compared to 0 μg/mL. **c** *P. aeruginosa* CCUG 51971 at 8 μg/mL compared to 0 μg/mL. **d** *P. aeruginosa* CCUG 70744 at 8 μg/mL compared to 0 μg/mL. **e** *P. aeruginosa* CCUG 70744 at 4 μg/mL compared to 0 μg/mL. **f** *P. aeruginosa* CCUG 70744 at 2 μg/mL compared to 0 μg/mL. The x-axis shows the log_2_ of the fold changes and the y-axis shows the -log_10_ of the *p*-values. Quantitated proteins are represented by dots. The colored squares represent the areas of significance, i.e., proteins within those areas had significantly downregulated or upregulated abundance levels (i.e., fold changes equal or higher than ±1.5 and *p*-values <0.05). The numbers within the colored squares indicate the number of significantly downregulated or upregulated proteins.

SUPPLEMENTARY TABLE 1 Number of proteins and peptides detected and quantitated in *Pseudomonas aeruginosa* strains CCUG 51971 and CCUG 70744.

SUPPLEMENTARY TABLE 2 List of proteins of *Pseudomonas aeruginosa* CCUG 51971 detected with more than one peptide. The table includes their relative abundances (sub-MICs vs. no antibiotic; if quantitated), basal relative abundance compared to strain PAO1 (if fulfilling the criteria for being included in the inter-strain the comparison, i.e., ≥99% of sequence identity and with 100% coverage over the longer protein), *P. aeruginosa* PAO1 orthologues and functional annotations. Orthologous proteins differentially regulated in both strains are in bold and shaded. Gene expression data from the publicly available transcriptomic dataset that analyzed biofilms of *P. aeruginosa* PAO1 treated with meropenem (5 μg/ml) vs. no antibiotic (GEO accession number: GSE167137) is included in columns AA – AC.

SUPPLEMENTARY TABLE 3 List of proteins of *Pseudomonas aeruginosa* CCUG 70744 detected with more than one peptide. The table includes their relative abundances (sub-MICs vs. no antibiotic; if quantitated), basal relative abundance compared to strain PAO1 (if fulfilling the criteria for being included in the inter-strain the comparison, i.e., ≥99% of sequence identity and with 100% coverage over the longer protein), *P. aeruginosa* PAO1 orthologues and functional annotations. Orthologous proteins differentially regulated in both strains are in bold and shaded. Gene expression data from the publicly available transcriptomic dataset that analyzed biofilms of *P. aeruginosa* PAO1 treated with meropenem (5 μg/ml) vs. no antibiotic (GEO accession number: GSE167137) is included in columns AA – AC.

SUPPLEMENTARY TABLE 4 List of regulatory proteins of *Pseudomonas aeruginosa* CCUG 51971 that were differentially regulated in at least one sub-MIC. The table includes their relative abundances (sub-MICs vs. no antibiotic; if quantitated), basal relative abundance compared to strain PAO1 (if fulfilling the criteria for being included in the inter-strain comparison, i.e., ≥99% of sequence identity and with 100% coverage over the longer protein), *P. aeruginosa* PAO1 orthologues and functional annotations. Orthologous proteins differentially regulated in both strains are in bold and shaded. Gene expression data from the publicly available transcriptomic dataset that analyzed biofilms of *P. aeruginosa* PAO1 treated with meropenem (5 μg/ml) vs. no antibiotic (GEO accession number: GSE167137) is included in columns AA – AC.

SUPPLEMENTARY TABLE 5 List of regulatory proteins of *Pseudomonas aeruginosa* CCUG 70744 that were differentially regulated in at least one sub-MIC. The table includes their relative abundances (sub-MICs vs. no antibiotic; if quantitated), basal relative abundance compared to strain PAO1 (if fulfilling the criteria for being included in the inter-strain comparison, i.e., ≥99% of sequence identity and with 100% coverage over the longer protein), *P. aeruginosa* PAO1 orthologues and functional annotations. Orthologous proteins differentially regulated in both strains are in bold and shaded. Gene expression data from the publicly available transcriptomic dataset that analyzed biofilms of *P. aeruginosa* PAO1 treated with meropenem (5 μg/ml) vs. no antibiotic (GEO accession number: GSE167137) is included in columns AA – AC.

SUPPLEMENTARY TABLE 6 List of proteins of *Pseudomonas aeruginosa* CCUG 51971 annotated as “hypothetical proteins” or as proteins with domain of unknown function in RefSeq that were differentially regulated in at least one sub-MIC. The table includes their relative abundances (sub-MICs vs. no antibiotic; if quantitated), basal relative abundance compared to strain PAO1 (if fulfilling the criteria for being included in the inter-strain comparison, i.e., ≥99% of sequence identity and with 100% coverage over the longer protein), *P. aeruginosa* PAO1 orthologues and functional annotations. Orthologous proteins differentially regulated in both strains are in bold and shaded. Gene expression data from the publicly available transcriptomic dataset that analyzed biofilms of *P. aeruginosa* PAO1 treated with meropenem (5 μg/ml) vs. no antibiotic (GEO accession number: GSE167137) is included in columns AA – AC.

SUPPLEMENTARY TABLE 7 List of proteins of *Pseudomonas aeruginosa* CCUG 70744 annotated as “hypothetical proteins” or as proteins with domain of unknown function in RefSeq that were differentially regulated in at least one sub-MIC. The table includes their relative abundances (sub-MICs vs. no antibiotic; if quantitated), basal relative abundance compared to strain PAO1 (if fulfilling the criteria for being included in the inter-strain comparison, i.e., ≥99% of sequence identity and with 100% coverage over the longer protein), *P. aeruginosa* PAO1 orthologues and functional annotations. Orthologous proteins differentially regulated in both strains are in bold and shaded. Gene expression data from the publicly available transcriptomic dataset that analyzed biofilms of *P. aeruginosa* PAO1 treated with meropenem (5 μg/ml) vs. no antibiotic (GEO accession number: GSE167137) is included in columns AA – AC.

SUPPLEMENTARY TABLE 8 Proteins of the H1-T6SS of *Pseudomonas aeruginosa* CCUG 51971. The table includes their relative abundances (sub-MICs vs. no antibiotic; if quantitated), basal relative abundance compared to strain PAO1 (if fulfilling the criteria for being included in the inter-strain comparison, i.e., ≥99% of sequence identity and with 100% coverage over the longer protein), *P. aeruginosa* PAO1 orthologues and functional annotation. Gene expression data from the publicly available transcriptomic dataset that analyzed biofilms of *P. aeruginosa* PAO1 treated with meropenem (5 μg/ml) vs. no antibiotic (GEO accession number: GSE167137) is included in columns AA – AC.

## Abbreviations

ABC: ATP-binding cassette
ANIb: Average nucleotide identity based on BLAST
bRP-LC: Basic reversed-phase liquid chromatography
CARD: Comprehensive Antibiotic Resistance Database
CCUG: Culture Collection University of Gothenburg
CFU: Colony forming unit
CID: Collision-induced dissociation
COG: Cluster of Orthologous Groups
DUF: Domain of unknown function
EC: Enzyme Commission
EDTA: Ethylenediaminetetraacetic acid
EUCAST: European Committee on Antimicrobial Susceptibility Testing
FASP: Filter-aided sample preparation
FDR: False discovery rate
GEO: Gene Expression Omnibus
GO: Gene Onthology
GSEA: Gene Set Enrichment Analysis
HCD: Higher-energy collisional dissociation
ICE: Integrative and conjugative element
KEGG: Kyoto Encyclopedia of Genes and Genomes
LC–MS/MS: Liquid chromatography tandem-mass spectrometry
MBL: Metallo-β-lactamase
MIC: Minimal inhibitory concentration
MLST: Multilocus sequence typing
NES: Normalized enrichment score
PBP: Penicillin-binding protein
PBS: Phosphate-buffered saline
PC: Principal component
PCA: Principal component analysis
PDC: *Pseudomonas*-derived cephalosporinase
PGAP: Prokaryotic Genome Annotation Pipeline
PSM: Peptide-spectrum match
RGI: Resistance Gene Identifier
RND: Resistance-nodulation-cell division
SDC: Sodium deoxycholate
SDS: Sodium dodecyl sulfate
ST: Sequence type
Sub-MIC: Subminimal inhibitory concentration
T6SS: Type VI secretion system
TEAB: Triethylammonium bicarbonate
TMT: Tandem mass tag

## References

[ref1] Alcock B. P. Raphenya A. R. Lau T. T. Y. Tsang K. K. Bouchard M. Edalatmand A.. (2020). CARD 2020: antibiotic resistome surveillance with the comprehensive antibiotic resistance database. Nucleic Acids Res. 48, D517–D525. doi: 10.1093/nar/gkz935, PMID: 31665441 PMC7145624

[ref2] Alfonso-Garrido J. Garcia-Calvo E. Luque-Garcia J. L. (2015). Sample preparation strategies for improving the identification of membrane proteins by mass spectrometry. Anal. Bioanal. Chem. 407, 4893–4905. doi: 10.1007/s00216-015-8732-0 25967148

[ref3] Aliashkevich A. Cava F. (2022). LD-transpeptidases: the great unknown among the peptidoglycan cross-linkers. FEBS J. 289, 4718–4730. doi: 10.1111/febs.16066 34109739

[ref4] Alvarez-Ortega C. Wiegand I. Olivares J. Hancock R. E. W. Martínez J. L. (2010). Genetic determinants involved in the susceptibility of *Pseudomonas aeruginosa* to β-lactam antibiotics. Antimicrob. Agents Chemother. 54, 4159–4167. doi: 10.1128/AAC.00257-10 20679510 PMC2944606

[ref5] Armengaud J. Christie-Oleza J. A. Clair G. Malard V. Duport C. (2012). Exoproteomics: exploring the world around biological systems. Expert Rev. Proteomics 9, 561–575. doi: 10.1586/epr.12.52, PMID: 23194272

[ref6] Armengaud J. Trapp J. Pible O. Geffard O. Chaumot A. Hartmann E. M. (2014). Non-model organisms, a species endangered by proteogenomics. J. Proteomics 105, 5–18. doi: 10.1016/j.jprot.2014.01.007 24440519

[ref7] Ashburner M. Ball C. A. Blake J. A. Botstein D. Butler H. Cherry J. M.. (2000). Gene ontology: tool for the unification of biology. Nat. Genet. 25, 25–29. doi: 10.1038/75556 10802651 PMC3037419

[ref8] Aubert D. Poirel L. Ben Ali A. Goldstein F. W. Nordmann P. (2001). OXA-35 is an OXA-10-related β-lactamase from *Pseudomonas aeruginosa*. J. Antimicrob. Chemother. 48, 717–721. doi: 10.1093/jac/48.5.717, PMID: 11679562

[ref9] Bagge N. Schuster M. Hentzer M. Ciofu O. Givskov M. Greenberg E. P.. (2004). *Pseudomonas aeruginosa* biofilms exposed to imipenem exhibit changes in global gene expression and β-lactamase and alginate production. Antimicrob. Agents Chemother. 48, 1175–1187. doi: 10.1128/AAC.48.4.1175-1187.2004 15047518 PMC375275

[ref10] Baquero F. (2001). Low-level antibacterial resistance: a gateway to clinical resistance. Drug Resist. Updat. 4, 93–105. doi: 10.1054/drup.2001.0196 11512526

[ref11] Barakat M. Ortet P. Whitworth D. E. (2013). P2RP: a web-based framework for the identification and analysis of regulatory proteins in prokaryotic genomes. BMC Genomics 14:269. doi: 10.1186/1471-2164-14-269 23601859 PMC3637814

[ref12] Bassetti M. Taramasso L. Giacobbe D. R. Pelosi P. (2012). Management of ventilator-associated pneumonia: epidemiology, diagnosis and antimicrobial therapy. Expert Rev. Anti Infect. Ther. 10, 585–596. doi: 10.1586/eri.12.36 22702322

[ref13] Bassetti M. Vena A. Croxatto A. Righi E. Guery B. (2018). How to manage *Pseudomonas aeruginosa* infections. Drugs Context 7, 1–18. doi: 10.7573/dic.212527, PMID: 29872449 PMC5978525

[ref14] Blackburn N. T. Clarke A. J. (2002). Characterization of soluble and membrane-bound family 3 lytic transglycosylases from *Pseudomonas aeruginosa*. Biochemistry 41, 1001–1013. doi: 10.1021/bi011833k, PMID: 11790124

[ref15] Blair J. M. A. Webber M. A. Baylay A. J. Ogbolu D. O. Piddock L. J. V. (2015). Molecular mechanisms of antibiotic resistance. Nat. Rev. Microbiol. 13, 42–51. doi: 10.1038/nrmicro3380 25435309

[ref16] Blázquez J. Gómez-Gómez J.-M. Oliver A. Juan C. Kapur V. Martín S. (2006). PBP3 inhibition elicits adaptive responses in *Pseudomonas aeruginosa*. Mol. Microbiol. 62, 84–99. doi: 10.1111/j.1365-2958.2006.05366.x, PMID: 16956383

[ref17] Blum M. Chang H.-Y. Chuguransky S. Grego T. Kandasaamy S. Mitchell A.. (2020). The InterPro protein families and domains database: 20 years on. Nucleic Acids Res. 49, D344–D354. doi: 10.1093/nar/gkaa977, PMID: 33156333 PMC7778928

[ref18] Bonnot T. Gillard M. B. Nagel D. H. (2019). A simple protocol for informative visualization of enriched gene ontology terms. Bioprotocol 9:e3429. doi: 10.21769/BioProtoc.3429

[ref19] Buchfink B. Xie C. Huson D. H. (2015). Fast and sensitive protein alignment using DIAMOND. Nat. Methods 12, 59–60. doi: 10.1038/nmeth.3176 25402007

[ref20] Cavallari J. F. Lamers R. P. Scheurwater E. M. Matos A. L. Burrows L. L. (2013). Changes to its peptidoglycan-remodeling enzyme repertoire modulate β-lactam resistance in *Pseudomonas aeruginosa*. Antimicrob. Agents Chemother. 57, 3078–3084. doi: 10.1128/AAC.00268-13, PMID: 23612194 PMC3697359

[ref21] Chen W. Zhang Y.-M. Davies C. (2017). Penicillin-binding protein 3 is essential for growth of *Pseudomonas aeruginosa*. Antimicrob. Agents Chemother. 61, e01651–e01616. doi: 10.1128/AAC.01651-16 27821444 PMC5192123

[ref22] Chevalier S. Bouffartigues E. Bodilis J. Maillot O. Lesouhaitier O. Feuilloley M. G. J.. (2017). Structure, function and regulation of *Pseudomonas aeruginosa* porins. FEMS Microbiol. Rev. 41, 698–722. doi: 10.1093/femsre/fux020 28981745

[ref23] Clark S. T. Sinha U. Zhang Y. Wang P. W. Donaldson S. L. Coburn B.. (2019). Penicillin-binding protein 3 is a common adaptive target among *Pseudomonas aeruginosa* isolates from adult cystic fibrosis patients treated with β-lactams. Int. J. Antimicrob. Agents 53, 620–628. doi: 10.1016/j.ijantimicag.2019.01.009 30664925

[ref24] Conesa A. Gotz S. Garcia-Gomez J. M. Terol J. Talon M. Robles M. (2005). Blast2GO: a universal tool for annotation, visualization and analysis in functional genomics research. Bioinformatics 21, 3674–3676. doi: 10.1093/bioinformatics/bti610 16081474

[ref26] Cortes-Lara S. Barrio-Tofiño E. D. López-Causapé C. Oliver A. Martínez-Martínez L. Bou G.. (2021). Predicting *Pseudomonas aeruginosa* susceptibility phenotypes from whole genome sequence resistome analysis. Clin. Microbiol. Infect. 27, 1631–1637. doi: 10.1016/j.cmi.2021.05.011 34015532

[ref27] Crone S. Vives-Flórez M. Kvich L. Saunders A. M. Malone M. Nicolaisen M. H.. (2019). The environmental occurrence of *Pseudomonas aeruginosa*. APMIS 128, 220–231. doi: 10.1111/apm.13010 31709616

[ref28] Curran B. Jonas D. Grundmann H. Pitt T. Dowson C. G. (2004). Development of a multilocus sequence typing scheme for the opportunistic pathogen *Pseudomonas aeruginosa*. J. Clin. Microbiol. 42, 5644–5649. doi: 10.1128/jcm.42.12.5644-5649.2004 15583294 PMC535286

[ref29] Davies T. A. Shang W. Bush K. Flamm R. K. (2008). Affinity of doripenem and comparators to penicillin-binding proteins in *Escherichia coli* and *Pseudomonas aeruginosa*. Antimicrob. Agents Chemother. 52, 1510–1512. doi: 10.1128/aac.01529-07 18250190 PMC2292531

[ref30] De Coster W. D'Hert S. Schultz D. T. Cruts M. Van Broeckhoven C. (2018). NanoPack: visualizing and processing long read sequencing data. Bioinformatics 34, 2666–2669. doi: 10.1093/bioinformatics/bty149 29547981 PMC6061794

[ref31] Deutsch E. W. Bandeira N. Sharma V. Perez-Riverol Y. Carver J. J. Kundu D. J.. (2019). The ProteomeXchange consortium in 2020: enabling ‘big data’ approaches in proteomics. Nucleic Acids Res. 48, D1145–D1152. doi: 10.1093/nar/gkz984 PMC7145525 31686107

[ref32] Diggle S. P. Whiteley M. (2020). Microbe profile: *Pseudomonas aeruginosa*: opportunistic pathogen and lab rat. Microbiology 166, 30–33. doi: 10.1099/mic.0.000860 31597590 PMC7273324

[ref33] Döring G. Parameswaran I. G. Murphy T. F. (2011). Differential adaptation of microbial pathogens to airways of patients with cystic fibrosis and chronic obstructive pulmonary disease. FEMS Microbiol. Rev. 35, 124–146. doi: 10.1111/j.1574-6976.2010.00237.x 20584083

[ref34] Eren E. Vijayaraghavan J. Liu J. Cheneke B. R. Touw D. S. Lepore B. W.. (2012). Substrate specificity within a family of outer membrane carboxylate channels. PLoS Biol. 10:e1001242. doi: 10.1371/journal.pbio.1001242 22272184 PMC3260308

[ref35] Fajardo A. Hernando-Amado S. Oliver A. Ball G. Filloux A. Martinez J. L. (2014). Characterization of a novel Zn^2+^−dependent intrinsic imipenemase from *Pseudomonas aeruginosa*. J. Antimicrob. Chemother. 69, 2972–2978. doi: 10.1093/jac/dku267 25185138

[ref36] Fajardo A. Martínez-Martín N. Mercadillo M. Galán J. C. Ghysels B. Matthijs S.. (2008). The neglected intrinsic resistome of bacterial pathogens. PLoS One 3:e1619. doi: 10.1371/journal.pone.0001619, PMID: 18286176 PMC2238818

[ref37] Frawley E. R. Fang F. C. (2014). The ins and outs of bacterial iron metabolism. Mol. Microbiol. 93, 609–616. doi: 10.1111/mmi.12709 25040830 PMC4135372

[ref38] Freschi L. Vincent A. T. Jeukens J. Emond-Rheault J.-G. Kukavica-Ibrulj I. Dupont M.-J.. (2019). The *Pseudomonas aeruginosa* pan-genome provides new insights on its population structure, horizontal gene transfer, and pathogenicity. Genome Biol. Evol. 11, 109–120. doi: 10.1093/gbe/evy259 30496396 PMC6328365

[ref39] Galperin M. Y. Makarova K. S. Wolf Y. I. Koonin E. V. (2015). Expanded microbial genome coverage and improved protein family annotation in the COG database. Nucleic Acids Res. 43, D261–D269. doi: 10.1093/nar/gku1223 25428365 PMC4383993

[ref40] Garcia-Clemente M. de la Rosa D. Máiz L. Girón R. Blanco M. Olveira C.. (2020). Impact of *Pseudomonas aeruginosa* infection on patients with chronic inflammatory airway diseases. J. Clin. Med. 9:3800. doi: 10.3390/jcm9123800 33255354 PMC7760986

[ref25] Gene Ontology Consortium (2020). The Gene Ontology resource: enriching a GOld mine. Nucleic Acids Res. 49, D325–D334. doi: 10.1093/nar/gkaa1113, PMID: 33290552 PMC7779012

[ref41] Giansanti P. Tsiatsiani L. Low T. Y. Heck A. J. R. (2016). Six alternative proteases for mass spectrometry–based proteomics beyond trypsin. Nat. Protoc. 11, 993–1006. doi: 10.1038/nprot.2016.057 27123950

[ref42] Girlich D. Naas T. Nordmann P. (2004). Biochemical characterization of the naturally occurring oxacillinase OXA-50 of *Pseudomonas aeruginosa*. Antimicrob. Agents Chemother. 48, 2043–2048. doi: 10.1128/aac.48.6.2043-2048.2004, PMID: 15155197 PMC415580

[ref43] Giske C. G. Rylander M. Kronvall G. (2003). VIM-4 in a carbapenem-resistant strain of *Pseudomonas aeruginosa* isolated in Sweden. Antimicrob. Agents Chemother. 47, 3034–3035. doi: 10.1128/AAC.47.9.3034-3035.2003 12937022 PMC182619

[ref44] Goris J. Konstantinidis K. T. Klappenbach J. A. Coenye T. Vandamme P. Tiedje J. M. (2007). DNA-DNA hybridization values and their relationship to whole-genome sequence similarities. Int. J. Syst. Evol. Microbiol. 57, 81–91. doi: 10.1099/ijs.0.64483-0 17220447

[ref45] Gurevich A. Saveliev V. Vyahhi N. Tesler G. (2013). QUAST: quality assessment tool for genome assemblies. Bioinformatics 29, 1072–1075. doi: 10.1093/bioinformatics/btt086, PMID: 23422339 PMC3624806

[ref46] Hachani A. Allsopp L. P. Oduko Y. Filloux A. (2014). The VgrG proteins are “à la carte” delivery systems for bacterial Type VI effectors*. J. Biol. Chem. 289, 17872–17884. doi: 10.1074/jbc.M114.563429 24794869 PMC4067218

[ref47] Haider S. Pal R. (2013). Integrated analysis of transcriptomic and proteomic data. Curr. Genomics 14, 91–110. doi: 10.2174/1389202911314020003 24082820 PMC3637682

[ref48] Hirsch E. B. Tam V. H. (2010). Impact of multidrug-resistant *Pseudomonas aeruginosa* infection on patient outcomes. Expert Rev. Pharmacoecon. Outcomes Res. 10, 441–451. doi: 10.1586/erp.10.49 20715920 PMC3071543

[ref49] Ho B. T. Dong T. G. Mekalanos J. J. (2014). A view to a kill: the bacterial type VI secretion system. Cell Host Microbe 15, 9–21. doi: 10.1016/j.chom.2013.11.008, PMID: 24332978 PMC3936019

[ref50] Hood R. D. Singh P. Hsu F. Güvener T. Carl M. A. Trinidad R. R. S.. (2010). A type VI secretion system of *Pseudomonas aeruginosa* targets a toxin to bacteria. Cell Host Microbe 7, 25–37. doi: 10.1016/j.chom.2009.12.007 20114026 PMC2831478

[ref51] Hua X. Liu L. Fang Y. Shi Q. Li X. Chen Q.. (2017). Colistin resistance in *Acinetobacter baumannii* MDR-ZJ06 revealed by a multiomics approach. Front. Cell. Infect. Microbiol. 7:45. doi: 10.3389/fcimb.2017.00045, PMID: 28275586 PMC5319971

[ref52] Huang Y. Niu B. Gao Y. Fu L. Li W. (2010). CD-HIT suite: a web server for clustering and comparing biological sequences. Bioinformatics 26, 680–682. doi: 10.1093/bioinformatics/btq003 20053844 PMC2828112

[ref53] Huerta-Cepas J. Forslund K. Coelho L. P. Szklarczyk D. Jensen L. J. von Mering C.. (2017). Fast genome-wide functional annotation through orthology assignment by eggNOG-mapper. Mol. Biol. Evol. 34, 2115–2122. doi: 10.1093/molbev/msx148 28460117 PMC5850834

[ref54] Huerta-Cepas J. Szklarczyk D. Heller D. Hernández-Plaza A. Forslund S. K. Cook H.. (2019). eggNOG 5.0: a hierarchical, functionally and phylogenetically annotated orthology resource based on 5090 organisms and 2502 viruses. Nucleic Acids Res. 47, D309–D314. doi: 10.1093/nar/gky1085, PMID: 30418610 PMC6324079

[ref55] Hulsen T. de Vlieg J. Alkema W. (2008). BioVenn – a web application for the comparison and visualization of biological lists using area-proportional Venn diagrams. BMC Genomics 9:488. doi: 10.1186/1471-2164-9-488, PMID: 18925949 PMC2584113

[ref56] Ikuta K. S. Swetschinski L. R. Robles Aguilar G. Sharara F. Mestrovic T. Gray A. P.. (2022). Global mortality associated with 33 bacterial pathogens in 2019: a systematic analysis for the global burden of disease study 2019. Lancet. 400, 2221–2248. doi: 10.1016/S0140-6736(22)02185-7 36423648 PMC9763654

[ref57] Isabella V. M. Campbell A. J. Manchester J. Sylvester M. Nayar A. S. Ferguson K. E.. (2015). Toward the rational design of carbapenem uptake in *Pseudomonas aeruginosa*. Chem. Biol. 22, 535–547. doi: 10.1016/j.chembiol.2015.03.018 25910245

[ref58] Johnning A. Karami N. Tang Hallback E. Muller V. Nyberg L. Buongermino Pereira M.. (2018). The resistomes of six carbapenem-resistant pathogens - a critical genotype-phenotype analysis. Microb. Genom. 4:e000233. doi: 10.1099/mgen.0.000233 30461373 PMC6321870

[ref59] Jones P. Binns D. Chang H.-Y. Fraser M. Li W. McAnulla C.. (2014). InterProScan 5: genome-scale protein function classification. Bioinformatics 30, 1236–1240. doi: 10.1093/bioinformatics/btu031 24451626 PMC3998142

[ref60] Jorgenson M. A. Chen Y. Yahashiri A. Popham D. L. Weiss D. S. (2014). The bacterial septal ring protein RlpA is a lytic transglycosylase that contributes to rod shape and daughter cell separation in *Pseudomonas aeruginosa*. Mol. Microbiol. 93, 113–128. doi: 10.1111/mmi.12643 24806796 PMC4086221

[ref61] Juan C. Moyá B. Pérez J. L. Oliver A. (2006). Stepwise upregulation of the *Pseudomonas aeruginosa* chromosomal cephalosporinase conferring high-level β-lactam resistance involves three AmpD homologues. Antimicrob. Agents Chemother. 50, 1780–1787. doi: 10.1128/AAC.50.5.1780-1787.2006, PMID: 16641450 PMC1472203

[ref62] Kamath K. S. Krisp C. Chick J. Pascovici D. Gygi S. P. Molloy M. P. (2017). *Pseudomonas aeruginosa* proteome under hypoxic stress conditions mimicking the cystic fibrosis lung. J. Proteome Res. 16, 3917–3928. doi: 10.1021/acs.jproteome.7b00561 28832155

[ref63] Kanehisa M. Furumichi M. Sato Y. Ishiguro-Watanabe M. Tanabe M. (2020). KEGG: integrating viruses and cellular organisms. Nucleic Acids Res. 49, D545–D551. doi: 10.1093/nar/gkaa970 PMC7779016 33125081

[ref64] Kitzis M. D. Acar J. F. Gutmann L. (1989). Antibacterial activity of meropenem against gram-negative bacteria with a permeability defect and against staphylococci. J. Antimicrob. Chemother. 24, 125–132. doi: 10.1093/jac/24.suppl_A.125 2808204

[ref65] Kong K.-F. Jayawardena S. R. Indulkar S. D. I. del Puerto A. Koh C.-L. Høiby N.. (2005). *Pseudomonas aeruginosa* AmpR is a global transcriptional factor that regulates expression of AmpC and PoxB β-lactamases, proteases, quorum sensing, and other virulence factors. Antimicrob. Agents Chemother. 49, 4567–4575. doi: 10.1128/AAC.49.11.4567-4575.2005 16251297 PMC1280116

[ref66] Kong K.-F. Schneper L. Mathee K. (2010). Beta-lactam antibiotics: from antibiosis to resistance and bacteriology. APMIS 118, 1–36. doi: 10.1111/j.1600-0463.2009.02563.x 20041868 PMC2894812

[ref67] Kong J. Wang Y. Xia K. Zang N. Zhang H. Liang X. (2021). New insights into the antibacterial and quorum sensing inhibition mechanism of *Artemisia argyi* leaf extracts towards *Pseudomonas aeruginosa* PAO1. 3 Biotech 11:97. doi: 10.1007/s13205-021-02663-5 PMC7840821 33520583

[ref68] Larsen M. V. Cosentino S. Rasmussen S. Friis C. Hasman H. Marvig R. L.. (2012). Multilocus sequence typing of total-genome-sequenced bacteria. J. Clin. Microbiol. 50, 1355–1361. doi: 10.1128/jcm.06094-11 22238442 PMC3318499

[ref69] Leinonen R. Sugawara H. Shumway M. on behalf of the International Nucleotide Sequence Database Collaboration (2011). The Sequence Read Archive. Nucleic Acids Res. 39, D19–D21. doi: 10.1093/nar/gkq1019 21062823 PMC3013647

[ref70] Li W. Godzik A. (2006). Cd-hit: a fast program for clustering and comparing large sets of protein or nucleotide sequences. Bioinformatics 22, 1658–1659. doi: 10.1093/bioinformatics/btl158 16731699

[ref71] Li K. W. Gonzalez-Lozano M. A. Koopmans F. Smit A. B. (2020). Recent developments in data independent acquisition (DIA) mass spectrometry: application of quantitative analysis of the brain proteome. Front. Mol. Neurosci. 13:564446. doi: 10.3389/fnmol.2020.564446, PMID: 33424549 PMC7793698

[ref72] Li H. Luo Y.-F. Williams B. J. Blackwell T. S. Xie C.-M. (2012). Structure and function of OprD protein in *Pseudomonas aeruginosa*: from antibiotic resistance to novel therapies. Int. J. Med. Microbiol. 302, 63–68. doi: 10.1016/j.ijmm.2011.10.001, PMID: 22226846 PMC3831278

[ref73] Li X.-Z. Plésiat P. (2016). “Antimicrobial drug efflux pumps in *Pseudomonas aeruginosa*” in Efflux-mediated antimicrobial resistance in bacteria: Mechanisms, regulation and clinical implications. eds. Li X.-Z. Elkins C. A. Zgurskaya H. I. (Cham: Springer International Publishing), 359–400.

[ref74] Li J. Yao Y. Xu H. H. Hao L. Deng Z. Rajakumar K.. (2015). SecReT6: a web-based resource for type VI secretion systems found in bacteria. Environ. Microbiol. 17, 2196–2202. doi: 10.1111/1462-2920.12794 25640659

[ref75] Liao X. Hancock R. E. (1997). Susceptibility to beta-lactam antibiotics of *Pseudomonas aeruginosa* overproducing penicillin-binding protein 3. Antimicrob. Agents Chemother. 41, 1158–1161. doi: 10.1128/AAC.41.5.1158 9145889 PMC163870

[ref76] Liao S. Zhang Y. Pan X. Zhu F. Jiang C. Liu Q.. (2019). Antibacterial activity and mechanism of silver nanoparticles against multidrug-resistant *Pseudomonas aeruginosa*. Int. J. Nanomedicine 14, 1469–1487. doi: 10.2147/IJN.S191340 30880959 PMC6396885

[ref77] Liu M. Li X. Xie Y. Bi D. Sun J. Li J.. (2018). ICEberg 2.0: an updated database of bacterial integrative and conjugative elements. Nucleic Acids Res. 47, D660–D665. doi: 10.1093/nar/gky1123 PMC6323972 30407568

[ref78] Llamas M. A. Sparrius M. Kloet R. Jiménez C. R. Vandenbroucke-Grauls C. Bitter W. (2006). The heterologous siderophores ferrioxamine B and ferrichrome activate signaling pathways in *Pseudomonas aeruginosa*. J. Bacteriol. 188, 1882–1891. doi: 10.1128/JB.188.5.1882-1891.2006, PMID: 16484199 PMC1426570

[ref79] Lodge J. M. Minchin S. D. Piddock L. J. V. Busby S. J. W. (1990). Cloning, sequencing and analysis of the structural gene and regulatory region of the *Pseudomonas aeruginosa* chromosomal *ampC* β-lactamase. Biochem. J. 272, 627–631. doi: 10.1042/bj2720627, PMID: 2125210 PMC1149754

[ref80] Lopatkin A. J. Bening S. C. Manson A. L. Stokes J. M. Kohanski M. A. Badran A. H.. (2021). Clinically relevant mutations in core metabolic genes confer antibiotic resistance. Science 371:eaba0862. doi: 10.1126/science.aba0862, PMID: 33602825 PMC8285040

[ref81] Magiorakos A. P. Srinivasan A. Carey R. B. Carmeli Y. Falagas M. E. Giske C. G.. (2012). Multidrug-resistant, extensively drug-resistant and pandrug-resistant bacteria: an international expert proposal for interim standard definitions for acquired resistance. Clin. Microbiol. Infect. 18, 268–281. doi: 10.1111/j.1469-0691.2011.03570.x 21793988

[ref82] Martin D. W. Holloway B. W. Deretic V. (1993). Characterization of a locus determining the mucoid status of *Pseudomonas aeruginosa*: AlgU shows sequence similarities with a *Bacillus* sigma factor. J. Bacteriol. 175, 1153–1164. doi: 10.1128/jb.175.4.1153-1164.1993, PMID: 8432708 PMC193032

[ref83] Martin D. W. Schurr M. J. Yu H. Deretic V. (1994). Analysis of promoters controlled by the putative sigma factor AlgU regulating conversion to mucoidy in *Pseudomonas aeruginosa*: relationship to sigma E and stress response. J. Bacteriol. 176, 6688–6696. doi: 10.1128/jb.176.21.6688-6696.1994 7961422 PMC197026

[ref84] Masuda N. Sakagawa E. Satoshi O. Gotoh N. Tsujimoto H. Nishino T. (2000). Substrate specificities of MexAB-OprM, MexCD-OprJ, and MexXY-OprM efflux pumps in *Pseudomonas aeruginosa*. Antimicrob. Agents Chemother. 44, 3322–3327. doi: 10.1128/AAC.44.12.3322-3327.2000 11083635 PMC90200

[ref85] McGuffie B. A. Vallet-Gely I. Dove S. L. (2016). σ factor and anti-σ factor that control swarming motility and biofilm formation in *Pseudomonas aeruginosa*. J. Bacteriol. 198, 755–765. doi: 10.1128/JB.00784-15 PMC4810613 26620262

[ref86] Meletis G. (2016). Carbapenem resistance: overview of the problem and future perspectives. Therap. Adv. Infect. Dis. 3, 15–21. doi: 10.1177/2049936115621709 26862399 PMC4735501

[ref87] Meyer B. Papasotiriou D. G. Karas M. (2011). 100% protein sequence coverage: a modern form of surrealism in proteomics. Amino Acids 41, 291–310. doi: 10.1007/s00726-010-0680-6, PMID: 20625782

[ref88] Micek S. T. Wunderink R. G. Kollef M. H. Chen C. Rello J. Chastre J.. (2015). An international multicenter retrospective study of *Pseudomonas aeruginosa* nosocomial pneumonia: impact of multidrug resistance. Crit. Care 19:219. doi: 10.1186/s13054-015-0926-5 25944081 PMC4446947

[ref89] Mistry J. Chuguransky S. Williams L. Qureshi M. Salazar G. A. Sonnhammer E. L. L.. (2021). Pfam: the protein families database in 2021. Nucleic Acids Res. 49, D412–D419. doi: 10.1093/nar/gkaa913 33125078 PMC7779014

[ref90] Monahan L. G. Turnbull L. Osvath S. R. Birch D. Charles I. G. Whitchurch C. B. (2014). Rapid conversion of *Pseudomonas aeruginosa* to a spherical cell morphotype facilitates tolerance to carbapenems and penicillins but increases susceptibility to antimicrobial peptides. Antimicrob. Agents Chemother. 58, 1956–1962. doi: 10.1128/AAC.01901-13 24419348 PMC4023726

[ref91] Moradali M. F. Ghods S. Rehm B. H. (2017). *Pseudomonas aeruginosa* lifestyle: a paradigm for adaptation, survival, and persistence. Front. Cell. Infect. Microbiol. 7:39. doi: 10.3389/fcimb.2017.00039, PMID: 28261568 PMC5310132

[ref92] Morata L. Cobos-Trigueros N. Martínez José A. Soriano Á. Almela M. Marco F.. (2012). Influence of multidrug resistance and appropriate empirical therapy on the 30-day mortality rate of *Pseudomonas aeruginosa* bacteremia. Antimicrob. Agents Chemother. 56, 4833–4837. doi: 10.1128/AAC.00750-12 22751533 PMC3421866

[ref93] Morita Y. Tomida J. Kawamura Y. (2014). Responses of *Pseudomonas aeruginosa* to antimicrobials. Front. Microbiol. 4:422. doi: 10.3389/fmicb.2013.00422 24409175 PMC3884212

[ref94] Mougous J. D. Cuff M. E. Raunser S. Shen A. Zhou M. Gifford C. A.. (2006). A virulence locus of *Pseudomonas aeruginosa* encodes a protein secretion apparatus. Science 312, 1526–1530. doi: 10.1126/science.1128393 16763151 PMC2800167

[ref95] Moya B. Dötsch A. Juan C. Blázquez J. Zamorano L. Haussler S.. (2009). β-Lactam resistance response triggered by inactivation of a nonessential penicillin-binding protein. PLoS Pathog. 5:e1000353. doi: 10.1371/journal.ppat.1000353, PMID: 19325877 PMC2654508

[ref96] Murray C. J. L. Ikuta K. S. Sharara F. Swetschinski L. Robles Aguilar G. Gray A.. (2022). Global burden of bacterial antimicrobial resistance in 2019: a systematic analysis. Lancet 399, 629–655. doi: 10.1016/S0140-6736(21)02724-0, PMID: 35065702 PMC8841637

[ref97] Naas T. Oueslati S. Bonnin R. A. Dabos M. L. Zavala A. Dortet L.. (2017). Beta-lactamase database (BLDB) – structure and function. J. Enzyme Inhib. Med. Chem. 32, 917–919. doi: 10.1080/14756366.2017.1344235 28719998 PMC6445328

[ref98] Nde C. W. Jang H.-J. Toghrol F. Bentley W. E. (2008). Toxicogenomic response of *Pseudomonas aeruginosa* to ortho-phenylphenol. BMC Genomics 9:473. doi: 10.1186/1471-2164-9-473 18847467 PMC2577666

[ref99] Nikaido H. Hancock R. E. W. (1986). “Outer membrane permeability of *Pseudomonas aeruginosa*” in The bacteria, a treatise on structure and function. ed. Sokatch J. R. (Orlando, Florida: Academic Press, Inc.)

[ref100] O'Leary N. A. Wright M. W. Brister J. R. Ciufo S. Haddad D. McVeigh R.. (2016). Reference sequence (RefSeq) database at NCBI: current status, taxonomic expansion, and functional annotation. Nucleic Acids Res. 44, D733–D745. doi: 10.1093/nar/gkv1189 26553804 PMC4702849

[ref101] Oliver A. Mulet X. López-Causapé C. Juan C. (2015). The increasing threat of *Pseudomonas aeruginosa* high-risk clones. Drug Resist. Updat. 21-22, 41–59. doi: 10.1016/j.drup.2015.08.002 26304792

[ref102] Pang Z. Raudonis R. Glick B. R. Lin T. J. Cheng Z. (2019). Antibiotic resistance in *Pseudomonas aeruginosa*: mechanisms and alternative therapeutic strategies. Biotechnol. Adv. 37, 177–192. doi: 10.1016/j.biotechadv.2018.11.013 30500353

[ref103] Pappireddi N. Martin L. Wühr M. (2019). A review on quantitative multiplexed proteomics. Chembiochem 20, 1210–1224. doi: 10.1002/cbic.201800650 30609196 PMC6520187

[ref104] Papp-Wallace K. M. Endimiani A. Taracila M. A. Bonomo R. A. (2011). Carbapenems: past, present, and future. Antimicrob. Agents Chemother. 55, 4943–4960. doi: 10.1128/aac.00296-11 21859938 PMC3195018

[ref105] Park A. J. Krieger J. R. Khursigara C. M. (2016). Survival proteomes: the emerging proteotype of antimicrobial resistance. FEMS Microbiol. Rev. 40, 323–342. doi: 10.1093/femsre/fuv051 26790948

[ref106] Pelegrin A. C. Palmieri M. Mirande C. Oliver A. Moons P. Goossens H.. (2021). *Pseudomonas aeruginosa*: a clinical and genomics update. FEMS Microbiol. Rev. 45:fuab026. doi: 10.1093/femsre/fuab026, PMID: 33970247

[ref107] Pérez-Llarena F. J. Bou G. (2016). Proteomics as a tool for studying bacterial virulence and antimicrobial resistance. Front. Microbiol. 7:410. doi: 10.3389/fmicb.2016.00410 27065974 PMC4814472

[ref108] Perez-Riverol Y. Csordas A. Bai J. Bernal-Llinares M. Hewapathirana S. Kundu D. J.. (2019). The PRIDE database and related tools and resources in 2019: improving support for quantification data. Nucleic Acids Res. 47, D442–D450. doi: 10.1093/nar/gky1106, PMID: 30395289 PMC6323896

[ref109] Pier G. B. (2007). *Pseudomonas aeruginosa* lipopolysaccharide: a major virulence factor, initiator of inflammation and target for effective immunity. Int. J. Med. Microbiol. 297, 277–295. doi: 10.1016/j.ijmm.2007.03.012 17466590 PMC1994162

[ref110] Potron A. Poirel L. Nordmann P. (2015). Emerging broad-spectrum resistance in *Pseudomonas aeruginosa* and *Acinetobacter baumannii*: mechanisms and epidemiology. Int. J. Antimicrob. Agents 45, 568–585. doi: 10.1016/j.ijantimicag.2015.03.001, PMID: 25857949

[ref111] Potter S. C. Luciani A. Eddy S. R. Park Y. Lopez R. Finn R. D. (2018). HMMER web server: 2018 update. Nucleic Acids Res. 46, W200–W204. doi: 10.1093/nar/gky448, PMID: 29905871 PMC6030962

[ref112] Potvin E. Lehoux D. E. Kukavica-Ibrulj I. Richard K. L. Sanschagrin F. Lau G. W.. (2003). In vivo functional genomics of *Pseudomonas aeruginosa* for high-throughput screening of new virulence factors and antibacterial targets. Environ. Microbiol. 5, 1294–1308. doi: 10.1046/j.1462-2920.2003.00542.x 14641575

[ref113] Rajput A. Tsunemoto H. Sastry A. V. Szubin R. Rychel K. Chauhan S. M.. (2022a). Advanced transcriptomic analysis reveals the role of efflux pumps and media composition in antibiotic responses of *Pseudomonas aeruginosa*. Nucleic Acids Res. 50, 9675–9688. doi: 10.1093/nar/gkac743 36095122 PMC9508857

[ref114] Rajput A. Tsunemoto H. Sastry A. V. Szubin R. Rychel K. Sugie J.. (2022b). Machine learning from *Pseudomonas aeruginosa* transcriptomes identifies independently modulated sets of genes associated with known transcriptional regulators. Nucleic Acids Res. 50, 3658–3672. doi: 10.1093/nar/gkac187, PMID: 35357493 PMC9023270

[ref115] Richter M. Rosselló-Móra R. Oliver Glöckner F. Peplies J. (2016). JSpeciesWS: a web server for prokaryotic species circumscription based on pairwise genome comparison. Bioinformatics 32, 929–931. doi: 10.1093/bioinformatics/btv681 26576653 PMC5939971

[ref116] Rodríguez-Martínez J.-M. Poirel L. Nordmann P. (2009a). Extended-spectrum cephalosporinases in *Pseudomonas aeruginosa*. Antimicrob. Agents Chemother. 53, 1766–1771. doi: 10.1128/aac.01410-08 19258272 PMC2681535

[ref117] Rodríguez-Martínez J.-M. Poirel L. Nordmann P. (2009b). Molecular epidemiology and mechanisms of carbapenem resistance in *Pseudomonas aeruginosa*. Antimicrob. Agents Chemother. 53, 4783–4788. doi: 10.1128/aac.00574-09 19738025 PMC2772299

[ref118] Salvà-Serra F. Svensson-Stadler L. Busquets A. Jaén-Luchoro D. Karlsson R. Moore E R. B.. (2018). A protocol for extraction and purification of high-quality and quantity bacterial DNA applicable for genome sequencing: a modified version of the Marmur procedure. Protocol Exchange. doi: 10.1038/protex.2018.084

[ref119] Sana T. G. Berni B. Bleves S. (2016). The T6SSs of *Pseudomonas aeruginosa* strain PAO1 and their effectors: beyond bacterial-cell targeting. Front. Cell. Infect. Microbiol. 6:61. doi: 10.3389/fcimb.2016.00061 27376031 PMC4899435

[ref120] Sauvage E. Kerff F. Terrak M. Ayala J. A. Charlier P. (2008). The penicillin-binding proteins: structure and role in peptidoglycan biosynthesis. FEMS Microbiol. Rev. 32, 234–258. doi: 10.1111/j.1574-6976.2008.00105.x 18266856

[ref121] Sayers E. W. Cavanaugh M. Clark K. Ostell J. Pruitt K. D. Karsch-Mizrachi I. (2020). GenBank. Nucleic Acids Res. 48, D84–D86. doi: 10.1093/nar/gkz956 31665464 PMC7145611

[ref122] Skurnik D. Roux D. Cattoir V. Danilchanka O. Lu X. Yoder-Himes D. R.. (2013). Enhanced in vivo fitness of carbapenem-resistant *oprD* mutants of *Pseudomonas aeruginosa* revealed through high-throughput sequencing. Proc. Natl. Acad. Sci. 110, 20747–20752. doi: 10.1073/pnas.1221552110 24248354 PMC3870709

[ref123] Starnbach M. N. Lory S. (1992). The *fliA* (*rpoF*) gene of *Pseudomonas aeruginosa* encodes an alternative sigma factor required for flagellin synthesis. Mol. Microbiol. 6, 459–469. doi: 10.1111/j.1365-2958.1992.tb01490.x, PMID: 1560774

[ref124] Stover C. K. Pham X. Q. Erwin A. L. Mizoguchi S. D. Warrener P. Hickey M. J.. (2000). Complete genome sequence of *Pseudomonas aeruginosa* PAO1, an opportunistic pathogen. Nature 406, 959–964. doi: 10.1038/35023079 10984043

[ref125] Sung K. Chon J. Kweon O. Nho S. Kim S. Park M.. (2021). Dynamic adaptive response of *Pseudomonas aeruginosa* to clindamycin/rifampicin-impregnated catheters. Antibiotics 10:752. doi: 10.3390/antibiotics10070752, PMID: 34206280 PMC8300626

[ref126] Tacconelli E. Carrara E. Savoldi A. Harbarth S. Mendelson M. Monnet D. L.. (2018). Discovery, research, and development of new antibiotics: the WHO priority list of antibiotic-resistant bacteria and tuberculosis. Lancet Infect. Dis. 18, 318–327. doi: 10.1016/S1473-3099(17)30753-3 29276051

[ref127] Tatusova T. DiCuccio M. Badretdin A. Chetvernin V. Nawrocki E. P. Zaslavsky L.. (2016). NCBI prokaryotic genome annotation pipeline. Nucleic Acids Res. 44, 6614–6624. doi: 10.1093/nar/gkw569, PMID: 27342282 PMC5001611

[ref128] Tavares I. M. Jolly L. Pompeo F. Leitão J. H. Fialho A. M. Sá-Correia I.. (2000). Identification of the *Pseudomonas aeruginosa* *glmM* gene, encoding phosphoglucosamine mutase. J. Bacteriol. 182, 4453–4457. doi: 10.1128/JB.182.16.4453-4457.2000 10913078 PMC94616

[ref129] Thompson A. Schäfer J. Kuhn K. Kienle S. Schwarz J. Schmidt G.. (2003). Tandem mass tags: a novel quantification strategy for comparative analysis of complex protein mixtures by MS/MS. Anal. Chem. 75, 1895–1904. doi: 10.1021/ac0262560 12713048

[ref130] Vidaillac C. Chotirmall S. H. (2021). *Pseudomonas aeruginosa* in bronchiectasis: infection, inflammation, and therapies. Expert Rev. Respir. Med. 15, 649–662. doi: 10.1080/17476348.2021.1906225 33736539

[ref131] Vogne C. Ramos Aires J. Bailly C. Hocquet D. Plésiat P. (2004). Role of the multidrug efflux system MexXY in the emergence of moderate resistance to aminoglycosides among *Pseudomonas aeruginosa* isolates from patients with cystic fibrosis. Antimicrob. Agents Chemother. 48, 1676–1680. doi: 10.1128/AAC.48.5.1676-1680.2004 15105120 PMC400545

[ref132] Wang J. Wang J. Wang Y. Sun P. Zou X. Ren L.. (2019). Protein expression profiles in methicillin-resistant *Staphylococcus aureus* (MRSA) under effects of subminimal inhibitory concentrations of imipenem. FEMS Microbiol. Lett. 366:fnz195. doi: 10.1093/femsle/fnz195 31529016

[ref133] Whitney J. C. Beck C. M. Goo Y. A. Russell A. B. Harding B. N. De Leon J. A.. (2014). Genetically distinct pathways guide effector export through the type VI secretion system. Mol. Microbiol. 92, 529–542. doi: 10.1111/mmi.12571 24589350 PMC4049467

[ref134] Wick R. R. Judd L. M. Gorrie C. L. Holt K. E. (2017). Unicycler: resolving bacterial genome assemblies from short and long sequencing reads. PLoS Comput. Biol. 13:e1005595. doi: 10.1371/journal.pcbi.1005595, PMID: 28594827 PMC5481147

[ref135] Wiegand I. Hilpert K. Hancock R. E. (2008). Agar and broth dilution methods to determine the minimal inhibitory concentration (MIC) of antimicrobial substances. Nat. Protoc. 3, 163–175. doi: 10.1038/nprot.2007.521, PMID: 18274517

[ref136] Winsor G. L. Griffiths E. J. Lo R. Dhillon B. K. Shay J. A. Brinkman F. S. (2016). Enhanced annotations and features for comparing thousands of *Pseudomonas* genomes in the Pseudomonas genome database. Nucleic Acids Res. 44, D646–D653. doi: 10.1093/nar/gkv1227, PMID: 26578582 PMC4702867

[ref137] Wiśniewski J. R. Zougman A. Nagaraj N. Mann M. (2009). Universal sample preparation method for proteome analysis. Nat. Methods 6, 359–362. doi: 10.1038/nmeth.1322 19377485

[ref138] Wolden R. Pain M. Karlsson R. Karlsson A. Aarag Fredheim E. G. Cavanagh J. P. (2020). Identification of surface proteins in a clinical *Staphylococcus haemolyticus* isolate by bacterial surface shaving. BMC Microbiol. 20:80. doi: 10.1186/s12866-020-01778-8 32264835 PMC7137321

[ref139] Yadav A. K. Espaillat A. Cava F. (2018). Bacterial strategies to preserve Cell Wall integrity against environmental threats. Front. Microbiol. 9:2064. doi: 10.3389/fmicb.2018.02064 30233540 PMC6127315

[ref140] Yakhnina A. A. McManus H. R. Bernhardt T. G. (2015). The cell wall amidase AmiB is essential for *Pseudomonas aeruginosa* cell division, drug resistance and viability. Mol. Microbiol. 97, 957–973. doi: 10.1111/mmi.13077, PMID: 26032134 PMC4646093

[ref141] Yang Y. Bhachech N. Bush K. (1995). Biochemical comparison of imipenem, meropenem and biapenem: permeability, binding to penicillin-binding proteins, and stability to hydrolysis by β-lactamases. J. Antimicrob. Chemother. 35, 75–84. doi: 10.1093/jac/35.1.75 7768785

[ref142] Yero D. Díaz-Lobo M. Costenaro L. Conchillo-Solé O. Mayo A. Ferrer-Navarro M.. (2021). The *Pseudomonas aeruginosa* substrate-binding protein Ttg2D functions as a general glycerophospholipid transporter across the periplasm. Commun. Biol. 4:448. doi: 10.1038/s42003-021-01968-8 33837253 PMC8035174

[ref143] Yoon E.-J. Jeong S. H. (2021). Mobile carbapenemase genes in *Pseudomonas aeruginosa*. Front. Microbiol. 12:614058. doi: 10.3389/fmicb.2021.614058 33679638 PMC7930500

[ref144] Yoshimura F. Nikaido H. (1982). Permeability of *Pseudomonas aeruginosa* outer membrane to hydrophilic solutes. J. Bacteriol. 152, 636–642. doi: 10.1128/jb.152.2.636-642.1982, PMID: 6813310 PMC221510

[ref145] Zamorano L. Moyà B. Juan C. Mulet X. Blázquez J. Oliver A. (2014). The *Pseudomonas aeruginosa* CreBC two-component system plays a major role in the response to β-lactams, fitness, biofilm growth, and global regulation. Antimicrob. Agents Chemother. 58, 5084–5095. doi: 10.1128/aac.02556-14 24936599 PMC4135852

[ref146] Zhang L. Hinz A. J. Nadeau J.-P. Mah T.-F. (2011). *Pseudomonas aeruginosa* *tssC1* links type VI secretion and biofilm-specific antibiotic resistance. J. Bacteriol. 193, 5510–5513. doi: 10.1128/JB.00268-11, PMID: 21784934 PMC3187457

[ref147] Zhao W.-H. Hu Z.-Q. (2010). β-Lactamases identified in clinical isolates of *Pseudomonas aeruginosa*. Crit. Rev. Microbiol. 36, 245–258. doi: 10.3109/1040841X.2010.481763 20482453

[ref148] Zincke D. Balasubramanian D. Silver L. L. Mathee K. (2016). Characterization of a carbapenem-hydrolyzing enzyme, PoxB, in *Pseudomonas aeruginosa* PAO1. Antimicrob. Agents Chemother. 60, 936–945. doi: 10.1128/aac.01807-15, PMID: 26621621 PMC4750667

